# Interfacial chemical oxidative synthesis of multifunctional polyfluoranthene†
†Electronic supplementary information (ESI) available: The optimization of polymerization conditions, size distribution and morphology of the PFA particles, and solubility and DMSO solution in sunlight and in 365 nm UV of FA and PFA synthesized. See DOI: 10.1039/c4sc03890h



**DOI:** 10.1039/c4sc03890h

**Published:** 2015-01-21

**Authors:** Xin-Gui Li, Yaozu Liao, Mei-Rong Huang, Richard B. Kaner

**Affiliations:** a State Key Laboratory of Pollution Control and Resource Reuse , College of Environmental Science and Engineering , Tongji University , 1239 Si-Ping Road , Shanghai 200092 , China . Email: adamxgli@yahoo.com ; Email: huangmeirong@tongji.edu.cn ; Fax: +86-21-65983869 ; Tel: +86-21-69582104; b Department of Chemistry & Biochemistry , California NanoSystems Institute , University of California, Los Angeles , Los Angeles , California 90095 , USA . Email: kaner@chem.ucla.edu ; Fax: +1 310 206 4038 ; Tel: +1 310 825 5346

## Abstract

A novel polyfluoranthene (PFA) exhibiting strong visual fluorescence emission, a highly amplified quenching effect, and widely controllable electrical conductivity is synthesized by the direct cationic oxidative polymerization of fluoranthene in a dynamic interface between *n*-hexane and nitromethane containing fluoranthene and FeCl_3_, respectively. A full characterization of the molecular structure signifies that the PFAs have a degree of polymerization from 22–50 depending on the polymerization conditions. A polymerization mechanism at the interface of the hexane/nitromethane biphasic system is proposed. The conductivity of the PFA is tunable from 6.4 × 10^–6^ to 0.074 S cm^–1^ by doping with HCl or iodine. The conductivity can be significantly enhanced to 150 S cm^–1^ by heat treatment at 1100 °C in argon. A PFA-based chemosensor shows a highly selective sensitivity for Fe^3+^ detection which is unaffected by other common metal ions. The detection of Fe^3+^ likely involves the synergistic effect of well-distributed π-conjugated electrons throughout the PFA helical chains that function as both the fluorophore and the receptor units.

## Abstract 2

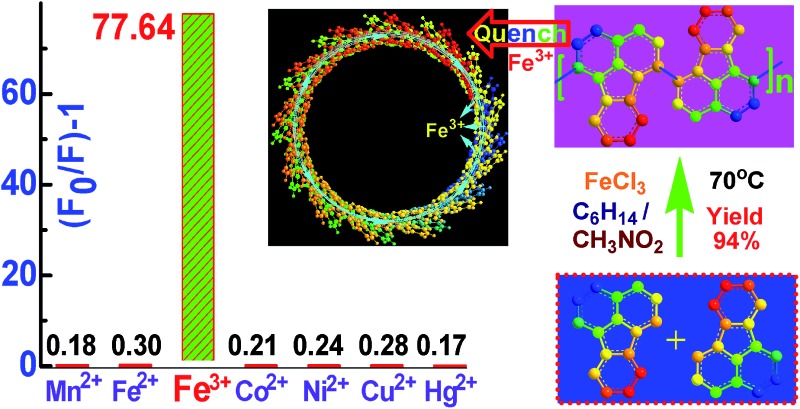
Polyfluoranthene, synthesized by an interfacial cationic oxidative polymerization of fluoranthene, emits visible fluorescence that shows highly selective sensitivity to Fe^3+^.

## Introduction

Conjugated aromatic polymers emit strong fluorescence that can be sensitively quenched by a specific substance.^1–5^ Fluoranthene (FA) possesses unique properties^6^ such as stronger fluorescence emission,^7,8^ longer fluorescence lifetime and higher fluorescence quantum yield than theoretically expected values,^9,10^ and has therefore found application as a dopant of molecular crystals for laser spectroscopy.^11,12^ It is believed that polyfluoranthene (PFA), with greater delocalized π-conjugation and higher molecular weight, could exhibit even better electronic and optoelectronic properties, higher carbon yield, and much lower toxicity than FA and even oligofluoranthene (OFA) consisting of 5 FA units.^12^ Moreover, since PFA contains repeat units of three hexa-carbocyclic rings and one penta-carbocyclic structure resulting in some gaps in its molecular chains, this could possibly make the polymer soluble or fusible. In fact, FA-based polymers have shown unique performance including low band gaps with novel electronic, optical, and photovoltaic properties due to their ladder topologies and highly delocalized π-conjugation.^13^ Up to the present, however, the synthesis, structure and properties of PFA have been little studied because FA has so high an oxidation potential that it could not be simply oxidized under common oxidizing conditions. Polymers comprising repeating units based on FA and its derivatives have been synthesized by Suzuki coupling reactions from 7,10-bis-(4-bromo-phenyl)-8,9-diphenyl-fluoranthene or 7,10-bis-(4-bromo-phenyl)-8,9-bis-(4-octyl-phenyl)-fluoranthene.^14^ However, the synthetic methodology applied to the derivatives and polymers of FA is rather sophisticated and costly. Electropolymerization has been successfully used to prepare PFA,^15,16^ but the resulting PFA films are structurally non-uniform and brittle, exhibiting low electrical conductivity,^15^ and poor solubility, thus preventing their processing and wider application. Additionally, the electrosynthesized PFA films have low thermal decomposition temperatures and char yields which are much lower than theoretically estimated values.^16^ In particular, the electropolymerization may also impede mass production since the synthetic productivity of PFA completely depends on the surface area of the noble metal electrode used.

In a previous study we reported that the homogeneous chemical oxidative polymerization of FA in nitromethane (CH_3_NO_2_) containing both FA and FeCl_3_ produces OFA with the ability to emit long-wavelength fluorescence at long excitation wavelengths along with high sensitivity to Fe^3+^ and picric acid. However, the drawbacks include relatively low synthetic yield, modest electrical conductivity, low thermal stability, and some toxicity.^12^ Here we demonstrate a novel method to efficiently synthesize PFA by an interfacial chemical oxidative polymerization of FA at a dynamic interface between *n*-hexane (C_6_H_14_) and CH_3_NO_2_ containing FA and FeCl_3_, respectively.^17^ The inspiration to directly synthesize PFA from FA originates from the specially π-conjugated system of FA. The synthesis, structure, properties, and functionalities of PFA were carefully explored and optimized by regulating synthetic parameters including the reaction medium, the method of mixing FA and FeCl_3_, the C_6_H_12_/CH_3_NO_2_ and FeCl_3_/FA ratios, polymerization temperature and time. Not only were the PFAs thoroughly characterized by using 13 modern investigative techniques, but the mechanism of the chemical oxidative polymerization of FA is also elucidated. A high-performance Fe^3+^ chemosensor possessing high selectivity, a wide linear range, and a superior detection limit for Fe^3+^ is demonstrated.

## Experimental section

### Interfacial chemical oxidative polymerization of FA at the C_6_H_14_/CH_3_NO_2_ interface

**Scheme 1:**
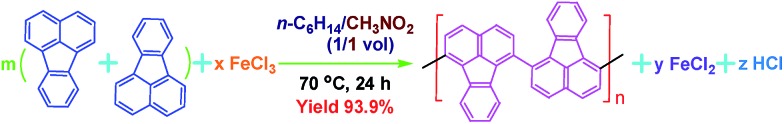
Chemical oxidative polymerization of FA in a biphasic interface between hexane and nitromethane.

In a typical synthesis of PFA, FA (417.0 mg, 2 mmol) was ultrasonically dissolved in 15 mL *n*-C_6_H_14_ in a 250 mL conical flask. Anhydrous FeCl_3_ oxidant (2270.8 mg, 14 mmol) was dissolved in 10 mL CH_3_NO_2_ in another 250 mL conical flask. After the oxidant solution was filtered to remove impurities, the monomer solution at 70 °C was added drop-wise into the purified oxidant solution at 70 °C within 15 min. The reactant solution was continually stirred magnetically at 70 °C for 18 h. During the polymerization, the formation of a bilayer reaction was observed, and the PFA particles formed changed their color from brownish red to dark blue and then to black. The PFA particles were collected from the polymerization medium and further purified with ethanol, 1.0 M HCl, 1.0 M NaOH, and DI water by centrifugation in order to respectively remove residual FA monomer, residual Fe^3+^, residual HCl, and residual NaOH until the upper liquid in the centrifuge tube was colorless. No Fe^3+^ residue in the PFA was confirmed by dropping aqueous K_4_Fe(CN)_6_ into the upper liquid, since no Prussian blue was observed. Finally, a fine dark PFA powder was obtained after drying in air at 50 °C for 48 h, giving an apparent polymerization yield of 93.9%. The nominal polymerization of FA is illustrated in Scheme 1.

### Enhancement of the conductivity of PFA particles

Three techniques including HCl doping, I_2_ vapor doping and carbonization have been used in attempts to enhance the conductivity of PFA particles, where the pristine PFA powder was previously heated at 80 °C for 48 h to remove *in situ* doped HCl. (1) For HCl doping, a 2 g L^–1^ dispersion of PFA in 4.0 M HCl was ultrasonically stirred for 24 h. HCl redoped PFA powder was obtained after filtration and drying at 40 °C for 48 h. (2) Following an early I_2_ doping technique,^17,18^ PFA powder and I_2_ particles were placed in a closed vessel but did not touch each other, and then were heated at a constant temperature of 80 °C under atmospheric pressure for 48 h. Note that the PFA powder became much darker or even black after I_2_ doping, but the PFA powder remained almost unchanged in a gray color before and after HCl doping. (3) The pristine PFA powder obtained under the optimal conditions was carbonized at a temperature up to 1100 °C by heating at a rate of 3–20 °C min^–1^ under nitrogen or argon atmosphere for *ca.* 1 h, and then cooled down to room temperature at 3 °C min^–1^. A black char was obtained with a char yield of 52–60 wt%.

### Characterization

UV-vis spectra of the PFA in NMP or 98% H_2_SO_4_ were obtained on a Perkin-Elmer Instruments Lambda 35 UV-vis spectrophotometer at a scanning rate of 400 nm min^–1^. ATR-FT-IR spectra were obtained with a Nicolet Magna-IRTM 550. Raman spectra of the solid powder of the PFA and the PFA-based char were achieved by British Renishaw inVia Raman Microscope (Bert) with a 785 nm red laser pumped by solid state diode. Elemental analysis was conducted using an Elementar Vario EL analyzer by Stephen Boyer at London Metropolitan University, UK. NMR spectra were obtained on Bruker spectrometers of AV700 for 1D ^1^H-NMR at 700 MHz, DQX400 for 2D ^1^H–^1^H COSY NMR at 400 MHz and AVC500 for ^13^C-NMR and ^1^H–^13^C HSQC at 500 MHz in DMSO-d6 at the University of Oxford, UK. The MALDI-TOF MS of the PFAs in THF with sinapinic acid as the matrix was recorded on a mass spectrometer by MALDI micro MX™ MICROMASS MS Technologies, Waters. Wide-angle X-ray diffraction patterns were scanned on a Philips X'pert Pro powder diffractometer using copper-monochromatized CuKα radiation (*λ* = 0.154178 nm). Fluorescence spectra were performed at the CNSI Advanced Light Microscopy/Spectroscopy Shared Facility at UCLA, USA. The quenching experiments of the fluorescence emission of 3 mL optimal PFA solution at 10 mg PFA/1 mL DMSO were carried out by adding 0.2 mL metal-ion aqueous solution. The melting point of FA and PFAs was determined using hot-stage optical microscopy. Simultaneous thermogravimetry and differential thermal analysis measurements were performed at a heating rate of 20 °C min^–1^ in nitrogen in a temperature up to 1000 °C and sample mass of 25 mg by using a NETZSCH STA 449C Jupiter thermogravimetric apparatus. The bulk electrical conductivity of an approximately 0.5 mm thick pressed disk of the PFA particles with an effective area of 0.785 cm^2^ was measured by a two-probe method.

## Results and discussion

### Optimal synthesis of PFA

**Fig. 1:**
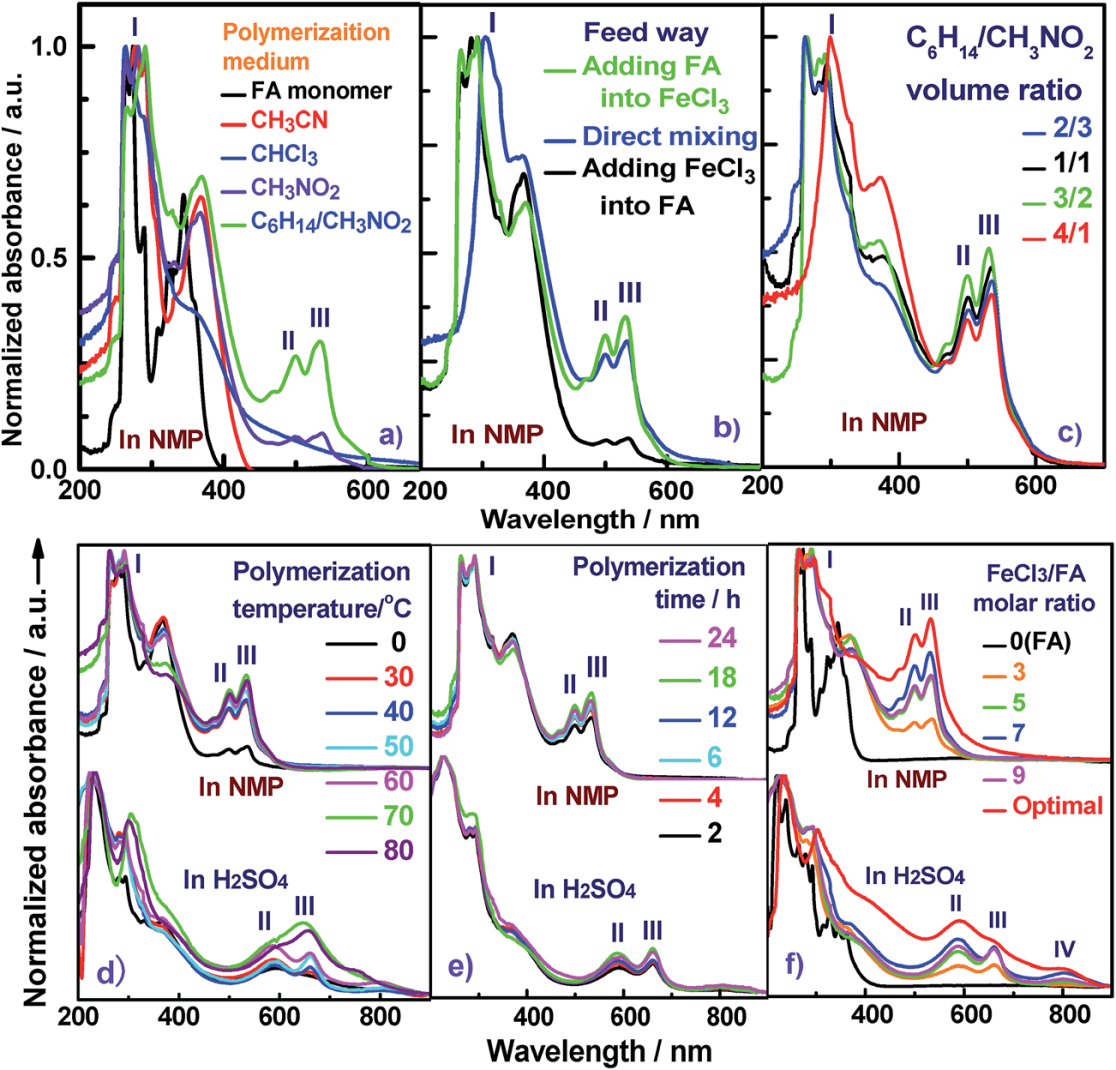
UV-vis spectra of PFA synthesized (a) in four different polymerization media; (b) by three different feed methods: (1) drop-wise addition of a FA solution to a FeCl_3_ solution, (2) direct mixing while holding the other conditions fixed (FeCl_3_/FA molar ratio of 5 in C_6_H_14_/CH_3_NO_2_ vol. ratio of 3/2 at 50 °C for 24 h), and (3) by drop-wise addition of a FeCl_3_ solution into a FA solution; and by (c) using four different C_6_H_14_/CH_3_NO_2_ volume ratios: 2/3, 1/1, 3/2 and 4/1, while holding the other conditions constant (drop-wise addition of a FA solution into FeCl_3_ solution and FeCl_3_/FA molar ratio of 7 at 70 °C for 24 h). The effect of (d) polymerization temperature at a fixed FeCl_3_/FA molar ratio of 5 for 24 h, (e) polymerization time at a fixed FeCl_3_/FA molar ratio of 5 at 50 °C, and (f) FeCl_3_/FA molar ratio at 50 °C for 18 h on UV-vis spectra of PFA.

Seven typical organic solvents including C_2_H_5_OH, CH_3_COOH, propylene carbonate, *n*-C_6_H_14_, CH_3_CN, CHCl_3_, CH_3_NO_2_, and an immiscible biphase of CH_3_NO_2_/C_6_H_14_ were examined as the medium for the chemical oxidative polymerization of FA. No dark polymer precipitates appear if C_2_H_5_OH, CH_3_COOH, propylene carbonate, or C_6_H_14_ are used as a single polymerization medium. A brown PFA precipitate was obtained in CH_3_CN or CHCl_3_, but Fig. 1a shows that the brown PFA does not exhibit any absorbance above 450 nm. Moreover, the brown PFA obtained in pure CH_3_NO_2_ shows a weak double peak at 500 and 534 nm that was not observed in the FA monomer. It appears that none of these seven solvents provide a suitable reaction medium for the synthesis of PFA with a satisfactorily large π-conjugated structure. Fortunately, the polymerization of FA in an immiscible biphasic solvent comprising CH_3_NO_2_/C_6_H_14_ can afford a black PFA that exhibits a much stronger double peak in exactly the same wavelength range from 500 and 534 nm, *i.e.*, this PFA product has a much larger π-conjugation length and/or higher molecular weight, significantly higher synthetic yield, considerably higher melting temperature, enhanced char yield at 1000 °C, and much higher conductivity than the OFA synthesized in CH_3_NO_2_ only,^12^ as summarized in Table 1. Considering (1) the high solubility of the FA monomer and FeCl_3_ in C_6_H_14_ and CH_3_NO_2_; (2) the moderate solubility of FA monomer in CH_3_NO_2_; and (3) the slight solubility of FeCl_3_ in C_6_H_14_, a slow but persistent interfacial polymerization between C_6_H_14_ and CH_3_NO_2_ is likely responsible for the formation of PFA with a large π-conjugation length. Conversely, the chain termination of FA polymerization with FeCl_3_ in a single solvent such as CH_3_NO_2_, CH_3_CN or CHCl_3_ may occur too early to form high molecular weight PFA. This is because early precipitation of growing macromolecular chains resulting from much lower solubility of PFA in the three single solvents will cause chain termination that can be avoided in CH_3_NO_2_/C_6_H_14_ as a result of better solubilization of FA and its active oligomers or intermediates. The novelty of the present study is that a new FA polymer having a new macromolecular structure and much higher performance has been achieved by a high-yield polymerization at the interface between CH_3_NO_2_ and C_6_H_14_ in an immiscible biphasic system. As a result, CH_3_NO_2_/C_6_H_14_ is considered as an ideal polymerization medium for the following investigation.

**Table 1:** Comparison of OFA and PFA synthesized in different media^*a*^

Polymerization media of FA	Product	Synthetic yield/%	Color	Solubility in NMP	Band III intensity	Exciting/emitting fluorescence wavelength/nm	Melting temperature/°C	Char yield at 1000 °C/wt%	Pristine	I_2_-doped
									Conductivity/S cm^–1^	
CH_3_NO_2_	OFA^12^	68.5	Brownish red	Soluble	0.085	395/494	299	29.0	<1.0 × 10^–9^	1.0 × 10^–4^
CH_3_NO_2_/C_6_H_14_	PFA	93.9	Brownish black	Mainly soluble	0.303	392/480	>380	60.3	6.4 × 10^–6^	7.4 × 10^–2^

^*a*^OFA and PFA were synthesized in CH_3_NO_2_ and an immiscible biphasic CH_3_NO_2_/C_6_H_14_ (1/1 vol) respectively with the same FeCl_3_/FA ratio of 5 at 50 °C for 24 h by drop-wise addition of FeCl_3_ into FA solutions. The FA monomer demonstrates exciting and emitting fluorescence wavelength of 365 and 474 nm, respectively.

The contact between monomer and oxidant during polymerization is one of the most critical parameters for the PFA synthesis. The polymerization of FA was conducted at a dynamic interface between CH_3_NO_2_ and C_6_H_14_ containing FeCl_3_ and FA, respectively, where a big difference of density exists (CH_3_NO_2_ (1.127 g mL^–1^) and C_6_H_14_ (0.659 g mL^–1^)). Three contact modes between monomer and oxidant were used in this study, including drop-wise addition of FA solution into FeCl_3_ solution, drop-wise addition of FeCl_3_ into FA, and direct mixing. In this study, all UV-vis spectra were normalized based on the strongest absorption of π-conjugated fused aromatic rings at 260–290 nm (band I), which was used as an internal standard in order to quantitatively analyze and compare the large π-conjugated structures of various PFAs. It is known that conjugated polymers possess more and longer π-conjugated structures if their UV-vis spectra exhibit stronger absorptions at longer wavelengths.^19^ In this way, the conjugation degree of PFA chains could be semi-quantitatively evaluated by the intensity ratio of the strongest band at 499–660 nm to band I at 287–310 nm.

It can be seen from Fig. 1b and Table S1† that the feed method for the FA and FeCl_3_ solutions significantly influences the polymerization of FA at a fixed reaction condition of C_6_H_14_/CH_3_NO_2_ volume ratio of 3/2 and FeCl_3_/FA molar ratio of 5 at 50 °C for 24 h. The drop-wise addition of FeCl_3_ solution into FA solution can produce PFA with the longest band III at a wavelength of 537 nm, and thus the longest π-conjugation, but the lowest polymerization yield of 60.5%, the smallest π-conjugation degree of 0.083, and the lightest yellow colored powder. In contrast, the drop-wise addition of FA solution into FeCl_3_ solution can achieve an optimal PFA demonstrating the highest polymerization yield of 72.4%, the darkest brown color, and the largest π-conjugation degree of 0.352 (Fig. 1b), regardless of its short band III wavelength of 533 nm. That is to say, the drop-wise addition of FA into FeCl_3_ is the best way of mixing the two reactants. Based on the densities of FeCl_3_ (2.898 g cm^–3^) and FA (1.252 g cm^–3^), it is obvious that the FeCl_3_/CH_3_NO_2_ solution is over twice as dense as the FA/C_6_H_14_ solution. When the addition of the dense FeCl_3_/CH_3_NO_2_ solution into the lighter FA/C_6_H_14_ solution was made, the redox polymerization between FeCl_3_ and FA did not satisfactorily progress because most oxidant rapidly sank to the bottom of the reaction vessel. In other words, the contact area between oxidant and monomer was insufficient to effectively produce PFA with a large degree of conjugation. Rapid direct mixing in one portion may cause dramatic polymerization in a short period of time, leading to insufficient conversion of FA and subsequently incomplete chain propagation. Fortunately, with the drop-wise addition of the lighter FA/C_6_H_14_ solution into the denser FeCl_3_/CH_3_NO_2_ solution, the suspended FA/C_6_H_14_ solution on the top of the oxidant solution not only creates enough space for interfacial controlled polymerization, but also provides adequate time for polymer chain propagation, ultimately resulting in the formation of PFA with a large π-conjugated structure. Therefore, the drop-wise addition of the light FA solution into the denser FeCl_3_ solution is an optimal feed method and therefore used for the following study.

The UV-vis absorption spectra of PFA obtained by four C_6_H_12_/CH_3_NO_2_ volume ratios at a fixed total volume of 25 mL are shown in Fig. 1c and Table S2.† These data show that the polymerization yield and large π-conjugation degree of the PFA both gradually increase at first and then rapidly decrease with increasing C_6_H_14_ fraction in the biphase media. The highest yield up to 88%, the strongest band III, and the highest degree of π-conjugation are simultaneously achieved for the PFA synthesized at a C_6_H_14_/CH_3_NO_2_ volume ratio of 3/2. The PFA obtained is also the darkest black. Clearly, the optimal C_6_H_14_/CH_3_NO_2_ volume ratio is 3/2 for the synthesis of PFA having the best comprehensive performance. Perhaps, too little C_6_H_14_ leads to a fast FA transfer into the CH_3_NO_2_ phase, thereby weakening the probability of interfacial polymerization, while too little CH_3_NO_2_ does not produce adequate interfacial area to effectively initiate polymerization. As a result, the C_6_H_14_/CH_3_NO_2_ volume ratio of 3/2 was selected as an optimal biphasic volume ratio for the following study.

**Fig. 2:**
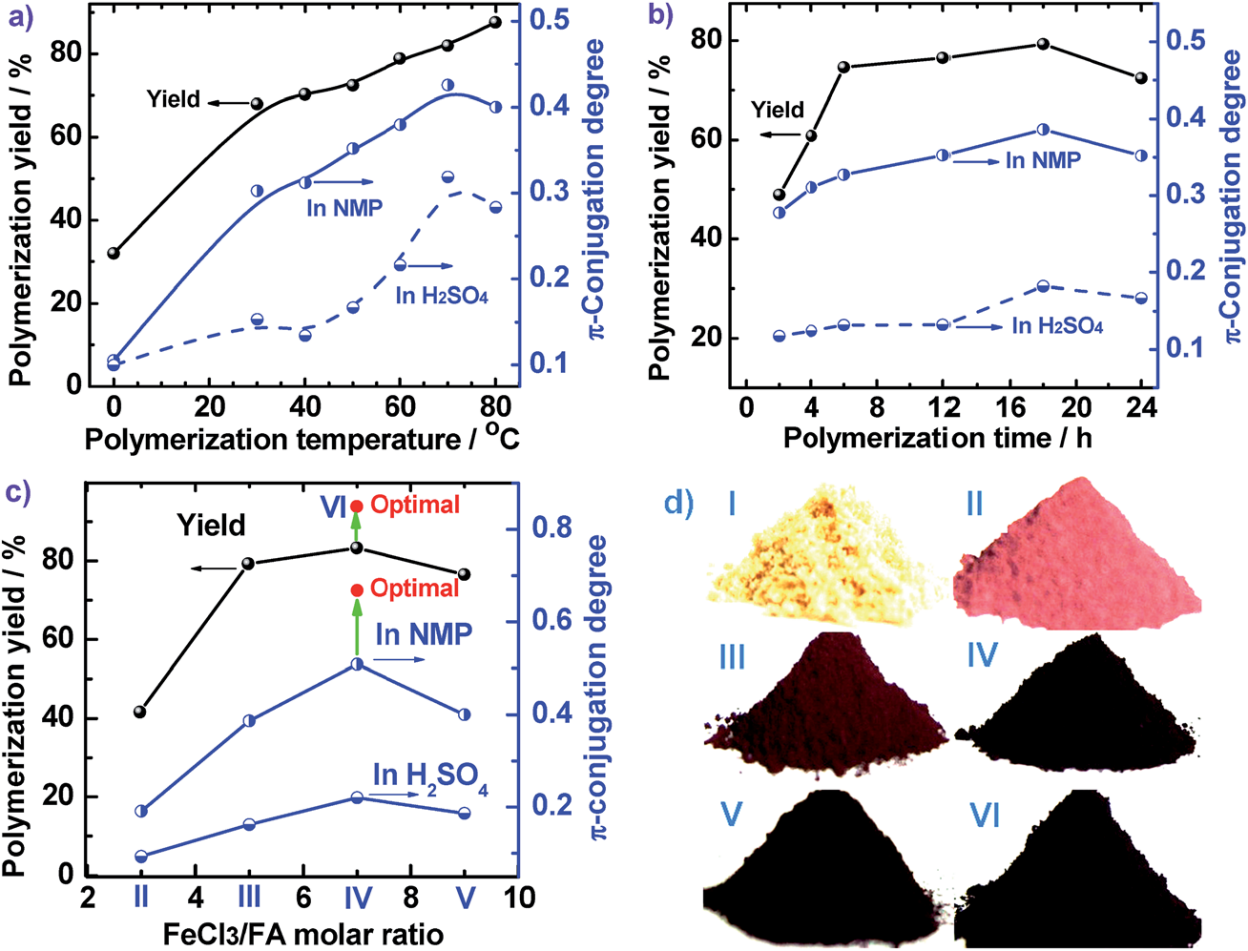
Effect of (a) polymerization temperature, (b) polymerization time, and (c) FeCl_3_/FA molar ratios on the polymerization yield and conjugation degree of PFA, and (d) the powder in sunlight of (I) FA and PFAs synthesized with various FeCl_3_/FA molar ratios: (II) 3, (III) 5, (IV) 7, (V) 9, and (VI) optimal PFA in C_6_H_14_/CH_3_NO_2_ as a biphasic medium with a volume ratio of 30 mL/20 mL at 50 °C.

The effect of polymerization temperature on the synthetic yield and conjugation degree of the PFA is demonstrated in Fig. 1d and 2a. By elevating the temperature from 0 to 80 °C at a fixed FeCl_3_/FA molar ratio of 5 for 24 h, the yield monotonically increases and reaches the highest value of 88% at 80 °C since high temperature can effectively activate the H-elimination reactivity on FA molecules and thereby result in efficient oxidative polymerization of FA. On the other hand, the contact area at the biphasic interface of C_6_H_14_/CH_3_NO_2_ could increase due to faster diffusion and more dramatic thermal motion of the reactants at higher temperature, thus enhancing the interaction of oxidants with monomers. This increase in the yield is opposite to significantly exothermic polymerizations of diaminonaphthalene,^20^ and diaminoanthraquinone,^21^ but matches the chemical oxidative polymerization of benzene,^22^ pyrene,^23^ and thiophene^17^ because they have similar dehydrogenation activity. However, the PFA in both solvents presents its maximum degree of π-conjugation at 70 °C, indicating that 70 °C is the best temperature to obtain PFA with the longest π-conjugated chains. Further elevation of the temperature is counter productive because a very high temperature accelerates polymerization, but also deteriorates the regularity of the PFA chain structure. Another reason is that the interface may be destroyed at too high a temperature because of the low boiling point of C_6_H_14_, although the reaction system was refluxed during the polymerization. Overall, 70 °C is the most suitable temperature for the synthesis of highly π-conjugated PFA, as discussed below.

Fig. 1e and 2b show the influence of polymerization time on the synthetic yield and π-conjugation degree of the PFA at a fixed FeCl_3_/FA molar ratio of 5 at 50 °C. With increasing polymerization time from 2 to 24 h, the polymerization yield and conjugation degree both rise steadily at first and subsequently decrease slightly. The maximum yield and degree of π-conjugation appear at a polymerization time of 18 h. A similar relationship between the time and polymerization yield is also found in the chemical oxidative polymerization of aniline.^24^ Generally, most chemical oxidative polymerizations have three typical reaction stages. In this case, first, FA monomers are quickly oxidized or initiated by FeCl_3_ and immediately converted into cationic radicals. Second, a strong interaction between the cationic radicals and monomers of FA lead to chain propagation, resulting in the formation of intermediate active PFA with high molecular weight. Third, the interaction between the intermediate active PFAs and/or FA monomers terminates the polymerization during the final stage of the polymerization. It is likely that increased polymerization time to 18 h is beneficial to chain propagation, but too long a polymerization time would cause an over oxidation of the PFA and thereby impair the yield and π-conjugation degree to some extent.

The influence of FeCl_3_ oxidant/FA monomer ratio on the synthetic yield and π-conjugation degree is summarized in Fig. 1f and 2c and Table S3† at fixed polymerization temperature and time of 50 °C and 24 h. It is observed that the yield and π-conjugation degree both rise at first and then decline as the FeCl_3_/FA molar ratio is increased from 3 to 9, demonstrating a maximum value at the FeCl_3_/FA molar ratio of 7. Note that the color of PFA powder gradually changed from light green (FA monomer) to brownish red, brown, dark black, and black after oxidation by the FeCl_3_/FA molar ratios of 3, 5, 7, and 9, respectively (Fig. 2d). That is, the PFA obtained at the FeCl_3_/FA molar ratio of 7 possesses the highest polymerization yield of 83%, the largest conjugation degree of 0.509, and the darkest black color. Lower yield and shorter conjugation length at lower oxidant/monomer ratios can be attributed to insufficient active sites for polymerization, while at a higher oxidant/monomer ratio of 9, an over-oxidation may take place, causing degradation of the PFA chains formed. It should be noted that the effect of FeCl_3_/FA ratio on the polymerization yield and π-conjugation degree of the PFA is weaker than that of temperature. Clearly, the optimal FeCl_3_/FA molar ratio is 7 for the synthesis of PFA with a maximum yield and highest degree of π-conjugation. As discussed above, the optimal condition for synthesizing PFA with the greatest conjugated structure is as follows: the drop-wise addition of FA solution to FeCl_3_ solution, C_6_H_12_/CH_3_NO_2_ volume ratio of 3/2, polymerization temperature of 70 °C, polymerization time of 18 h, and FeCl_3_/FA molar ratio of 7. PFA synthesized under these optimal conditions results in the highest polymerization yield of 93.9%, the largest degree of conjugation (0.668) in NMP, and the darkest black as can be seen in Fig. 1f, 2c and d(VI).

### Structure and morphology of PFA

The molecular structure of the synthesized PFA was systematically characterized by a combination of UV-vis, IR, Raman, elemental analysis, 2D ^1^H–^1^H COSY, 2D ^1^H–^13^C HSQC, ^13^C NMR, ^1^H NMR, GPC, and MALDI-MS techniques. Fig. 1f displays UV-vis spectra of FA and the soluble part of the optimal PFA. The FA monomer shows two major characteristic absorptions in the wavelength range from 200 to 400 nm,^25^ but no absorption bands at wavelengths longer than 400 nm. Although the PFA displays slightly different bands between 200 and 400 nm, it also demonstrates three new bands between 400 and 900 nm. These additional bands at longer wavelengths can be assigned to the large π-conjugated structure of PFA chains. Notably, the UV-vis spectra of PFA are quite different in each solvent, in which the bands between 400 and 900 nm are weaker and appear at longer wavelength in H_2_SO_4_ than in NMP. This suggests that the PFA exhibits significantly different chain conformations and doping levels. The weakest band at 800 nm in H_2_SO_4_ may be related to the extension of the large π-conjugated electrons along the macromolecular chains. It is surprising to discover that the PFA synthesized in this study has much stronger UV-vis bands than electropolymerized PFA,^9^ indicating that the PFA obtained by chemical oxidative polymerization of FA is a longer π-conjugated polymer with much higher molecular weight.

**Fig. 3:**
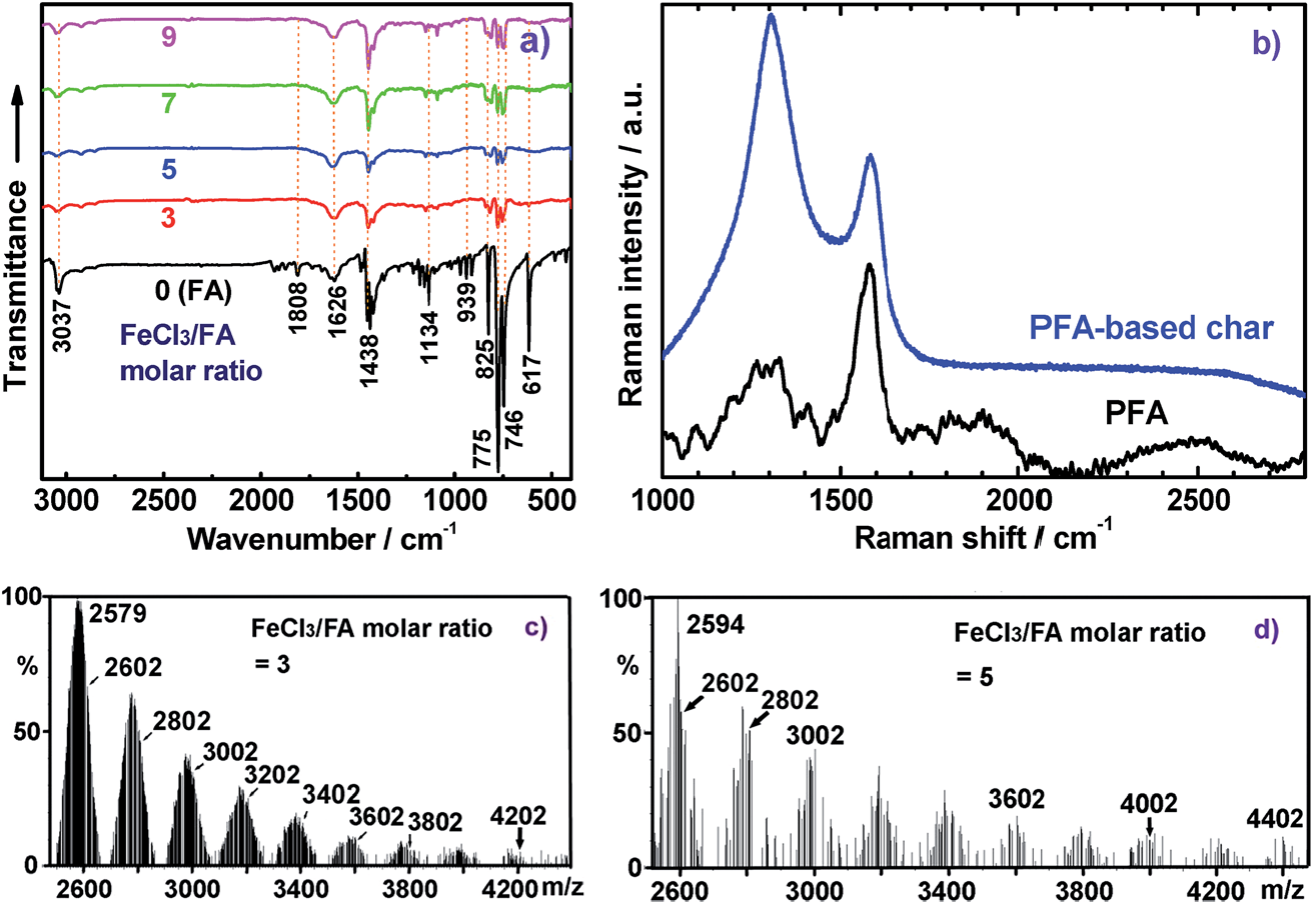
(a) IR spectra of FA monomer and PFA synthesized with various FeCl_3_/FA ratios at fixed conditions: drop-wise addition of FA monomer solution, C_6_H_14_/CH_3_NO_2_ volume ratio of 3/2, polymerization temperature of 50 °C, and polymerization time of 18 h. (b) Raman spectra of the PFA particles with FA/FeCl_3_ molar ratio of 7 and the PFA particles-based char at high temperature in nitrogen; (c–d) MALDI-TOF MS spectra of FA monomer and PFA synthesized with various FeCl_3_/FA ratios at fixed conditions: drop-wise addition of FA monomer solution, C_6_H_14_/CH_3_NO_2_ volume ratio of 3/2, polymerization temperature of 50 °C, and polymerization time of 18 h.

The IR spectra for FA monomer and dedoped PFA synthesized with four different FeCl_3_/FA ratios in the biphase system are presented in Fig. 3a, in which all the IR spectra were normalized to the intensity of the band at 1622 cm^–1^ due to C=C skeletal vibrations in the FA ring; this can be done since its framework would not be expected to change significantly after polymerization. Evidently, the IR spectra of dark colored PFA powders are quite different from that of the FA monomer, implying that PFA has a higher degree of π-conjugation with a higher molecular weight. For example, FA monomer exhibits a band at 3039 cm^–1^ owing to aromatic C–H stretching,^26^ which becomes weaker and broader in all of the PFAs and is hypsochromically shifted to 3049 cm^–1^. The zig–zag bands of FA monomer around 1808, 1134, and 939 cm^–1^ are barely observed in the IR spectra of PFAs because the small π-conjugation within the FA ring has converted into larger π-conjugation along the PFA chains after polymerization. The band at 1438 cm^–1^ due to C=C out-of-plane bending in FA monomer becomes weaker, but is hypsochromically shifted to 1452 cm^–1^ in PFA. In particular, several typical bands for aromatic compounds at 825, 775, and 746 cm^–1^ that are attributable to out-of-plane vibrations of adjacent C–H structures in the FA monomer,^27,28^ distinctly become much weaker and broader in the PFAs. All this information confirms the formation of PFA with a large π-conjugated structure and even higher molecular weight produced at the interface between C_6_H_14_ and CH_3_NO_2_. In addition, by increasing the FeCl_3_/FA molar ratio from 3 to 9, the IR spectra of PFAs change slightly; however, the 1452 cm^–1^ band is the strongest for the PFAs synthesized at the FeCl_3_/FA molar ratios of 7 and 9, suggesting that both PFAs have the largest degree of conjugation and the highest molecular weight, which is verified by Fig. 1f and 2c.

The Raman spectrum of the solid microparticles of PFA in Fig. 3b shows several characteristic Raman bands centered at 1581 (the strongest and sharpest), 1329, 1266, 1292, 1200, 1409, 1479, and 1097 cm^–1^ corresponding to the Raman vibration of FA units. Compared with FA monomers,^29^ these Raman bands generally shift to lower wavenumber, which provides further evidence for PFA formation.

Elemental analysis results in Table 2 have been used to help establish molecular structures for the PFA synthesized at the polymerization temperatures of 50 and 70 °C (optimal) at the fixed optimal conditions mentioned above. It can be calculated that the actual C/H weight ratio is 8.00/4.03 and 8.00/4.00 in the PFAs synthesized at 50 and 70 °C respectively. These two PFAs display the highest *M*_*m*/*z*_ of 4402 and 10 002 by MALDI/TOF MS as discussed below. The PFA synthesized at 50 °C has lower molecular mass than that at 70 °C since the former has stronger spatial hindrance leading to more difficult chain propagation. As a result, the proposed formulas and macromolecular structures in Table 2 are in good agreement with the experimental results. This suggests that both PFAs synthesized at 50 and 70 °C (optimal) have a helical type structure. This is further supported by the fact that the rotation of the “single bonds” linking repetitive FA units is heavily confined because (1) bulky FA units have a large spatial steric hindrance and (2) the single bonds actually have some double bond character resulting from the large π-conjugated structure along the whole PFA chains possessing intrinsic electrical conductivity of 6.4 × 10^–6^ and 7.4 × 10^–2^ S cm^–1^ for as-prepared and iodine doped PFAs, respectively.

**Table 2:** Elemental analyses of PFAs synthesized under optimal polymerization conditions

Polymerization temperature (°C)	50	70 (Optimal)
C/H/N (wt%)	92.18/4.15/0.10	82.34/3.42/0.18
C/H (mol%)	8.00/4.03	8.00/4.00
Formula	C_352_H_176_	C_800_H_400_
Macromolecular structure	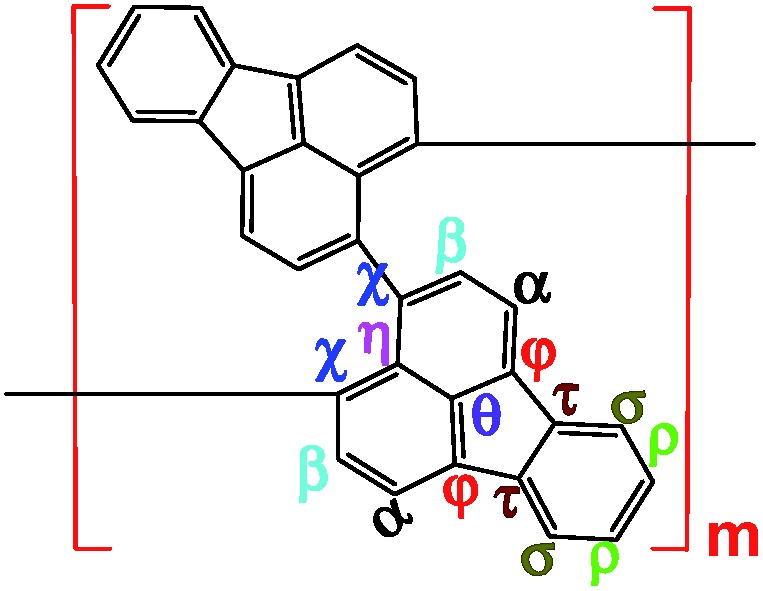	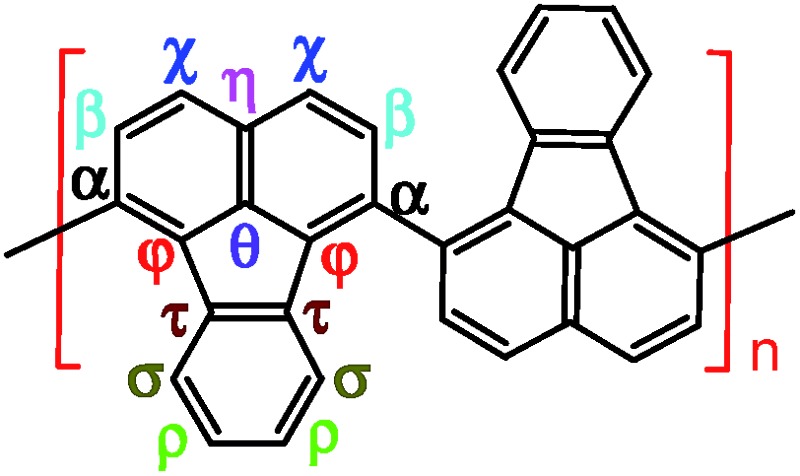
3D macromolecular structure emitting skyblue fluorescence (the helix spatial configurations of the ball and stick model of the PFA with the minimal energy were determined by Chem3D Ultra Molecular Modeling and analysis 2003)	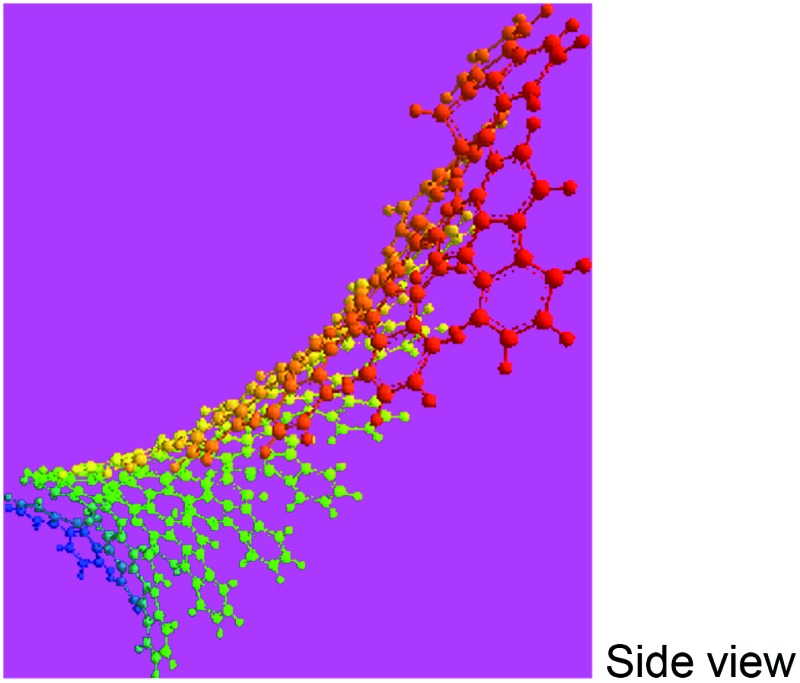	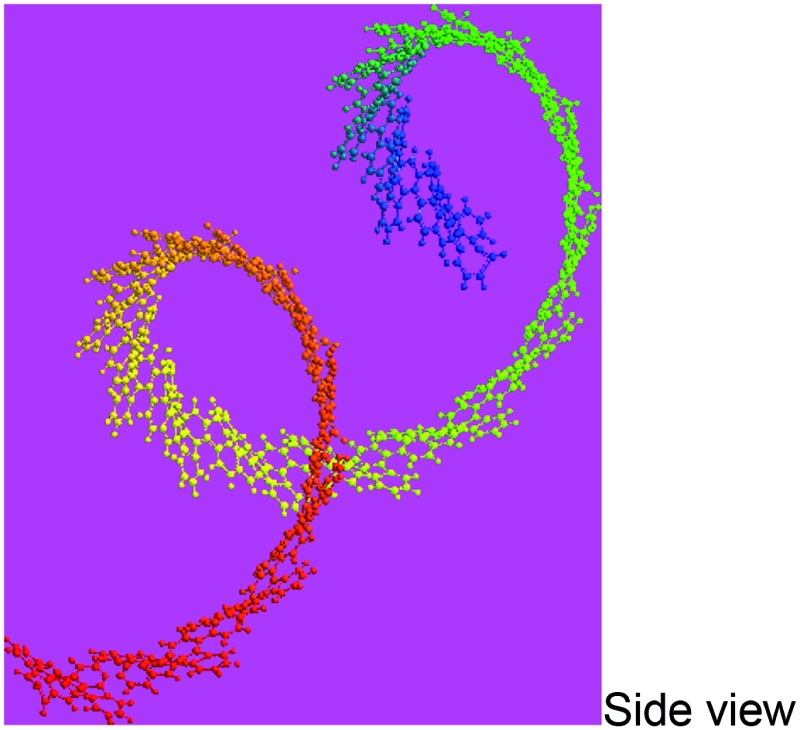
	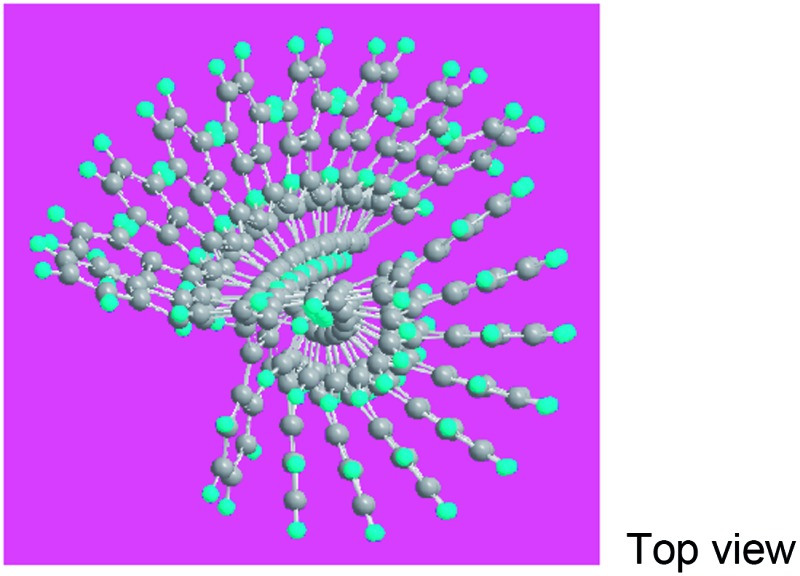	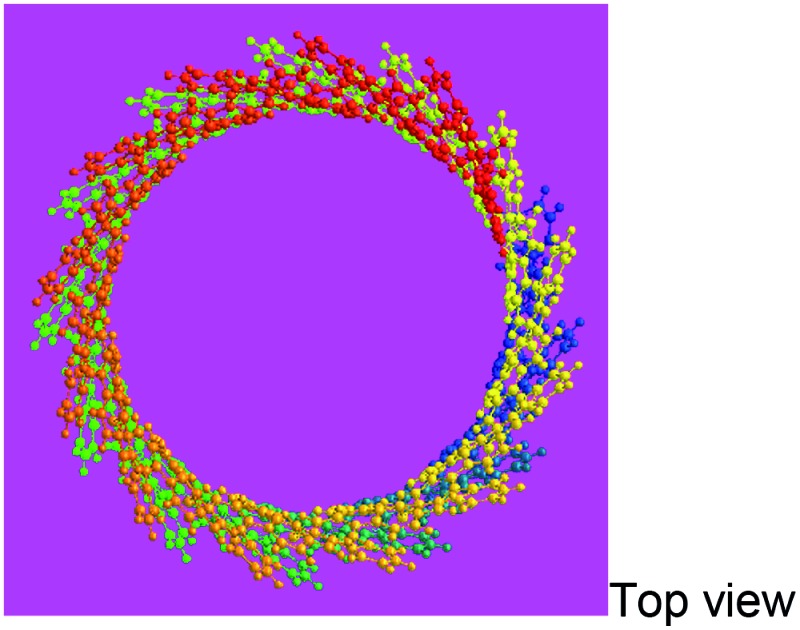

MALDI-TOF MS is a powerful method for characterizing synthetic polymers, giving the molecular weight of PFAs from 4202, 4402, 4202 to 4402 to 10 002 while changing the oxidant/monomer molar ratio from 3, 5, 7 to 9 to optimal, as shown in Fig. 3c and d of two representative PFAs. It seems that all of the PFAs consist of disubstituted fluoranthene repeat units except for the PFA obtained at the FeCl_3_/FA molar ratio of 9, which is a linear polymer. The molecular weight polydispersity index (PDI) of the THF-soluble parts of the PFAs determined by GPC varies from 1.882 to 1.907 to 1.980 to 1.869 to 1.705 when the oxidant/monomer molar ratio is changed from 3 to 5 to 7 to 9 to optimal. It can be concluded that the optimal PFA possesses the highest molecular weight, but the narrowest molecular weight distribution as confirmed by the UV-vis and NMR spectra above. In other words, the PFA achieved under the optimal conditions is really optimal.

**Fig. 4:**
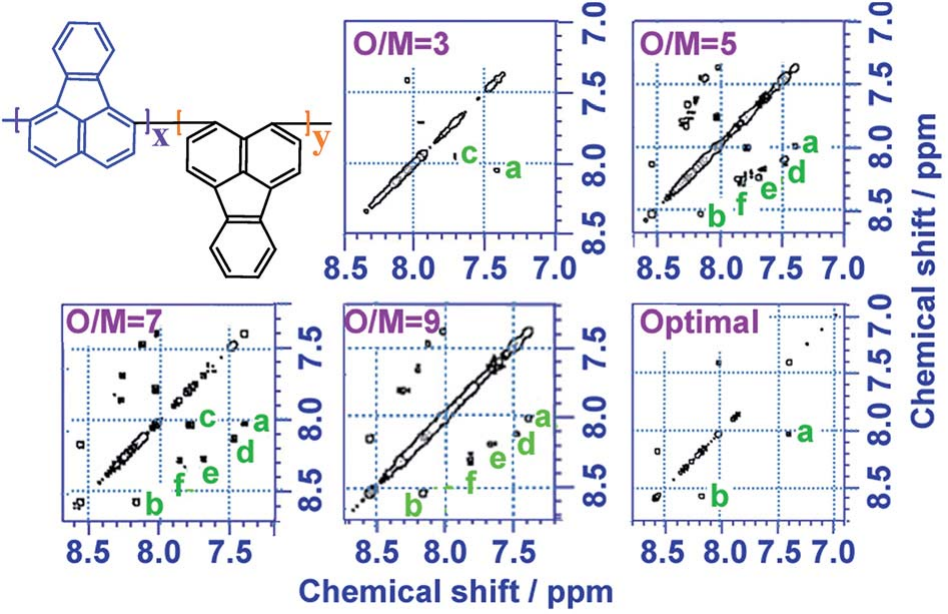
^1^H–^1^H 2D COSY spectra of PFA synthesized with various oxidant FeCl_3_/monomer FA (O/M) molar ratios of 3, 5, 7, 9, and optimal conditions; the upper left corner shows the proposed macromolecular structure.

^1^H–^1^H COSY and ^1^H–^13^C HSQC 2D NMR spectra are powerful methods of analyzing the complicated macromolecular structure of the PFAs containing a large number of very similar protons and carbons. As presented in Fig. 4, the ^1^H–^1^H COSY spectra significantly vary with the oxidant/monomer ratio, displaying six principle coupling peaks in a range between 7.0 and 9.0 ppm, (a) 7.42–8.04 ppm, (b) 8.18–8.58 ppm, (c) 7.72–8.00 ppm, (d) 7.49–8.10 ppm, (e) 7.70–8.20 ppm, and (f) 7.88–8.30 ppm. Interestingly, PFA synthesized at the lowest oxidant/monomer molar ratio of 3 only presents two correlations, *i.e.*, *a*(*ρ*7.42, *σ*8.04) and *c*(*β*7.72, *α*7.95), having 3,4-disubstituted fluoranthene repeat units (Scheme 1 and Table 2). The optimal PFA also displays one similar and one different correlation, *i.e.*, *a*(*ρ*7.42, *σ*8.03) and *b*(*β*8.18, *χ*8.58), having the 1,6-disubstituted fluoranthene repeat unit. When increasing the oxidant/monomer molar ratio from 3 to 7, the appearance of a series of new correlations (*e*, *d*, *e*, *f*) implies that the polymers have a mixed chain composition of 3,4- and 1,6-disubstituted fluoranthene units and even other disubstituted fluoranthene units, except that the polymer obtained at an oxidant/monomer molar ratio of 9 is similar to the optimal polymer rather than at an oxidant/monomer molar ratio of 3 because no *c* correlation is observed in the COSY spectrum of the polymer formed at an oxidant/monomer ratio of 9.

**Fig. 5:**
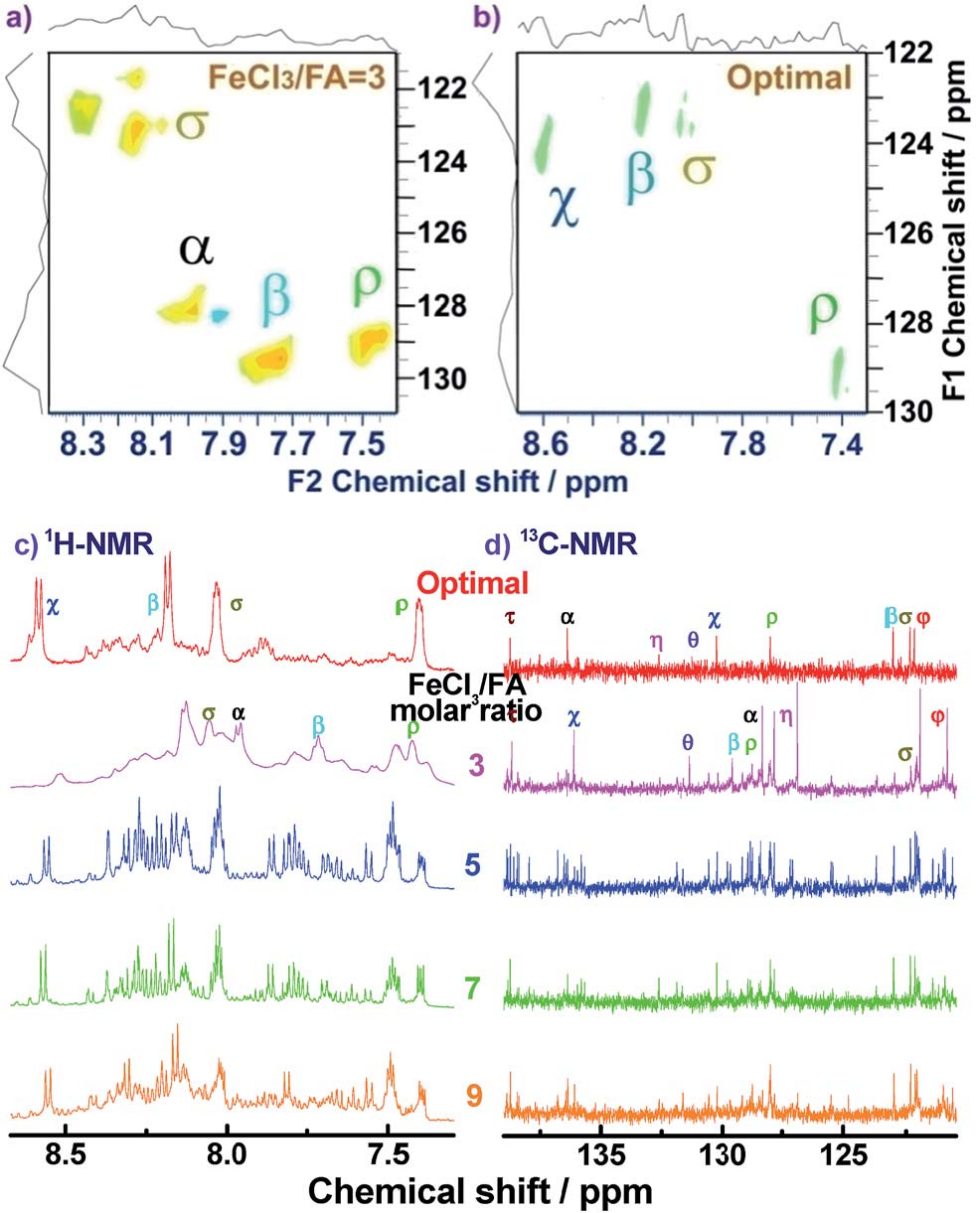
^1^H–^13^C 2D HSQC spectra of (a) PFA synthesized with an FeCl_3_/FA molar ratio of 3 at fixed polymerization temperature of 50 °C and polymerization time of 18 h and (b) PFA synthesized under the optimal conditions. (c) ^1^H-NMR and (d) ^13^C-NMR spectra of the PFAs synthesized with various FeCl_3_/FA molar ratios.

For the assignment of the ^13^C spectra of PFA, ^1^H–^13^C correlations are represented as a contour plot by HSQC experiments. The ^1^H–^13^C 2D HSQC spectra consist of several correlation resonances of only the carbons linking protons. According to the earlier discussion of chemical shifts of the protons in the ^1^H–^1^H 2D COSY spectra in Fig. 4, the ^1^H–^13^C correlations could be present in the HSQC spectra in Fig. 5a and b. The four major ^1^H–^13^C correlations of PFA synthesized with an oxidant/monomer molar ratio of 3 are assigned to *ρ*(H7.42, C129.0), *β*(H7.72, C129.5), *α*(H7.95, 128.0), *σ*(H8.04, C123.0). In contrast, the optimal PFA exhibits a different HSQC spectrum, *ρ*(H7.42, C129.0), *σ*(H8.03, C123.5), *β*(H8.18, C123.2), *χ*(H8.58, C124.3).

Combined with the ^1^H–^1^H COSY and ^1^H–^13^C HSQC 2D spectra, possible assignments of other aromatic protons and carbons in the polymers are depicted in Fig. 5c and d. The chemical shifts of the four main protons in optimal PFA have been assigned above, and then the chemical shifts of nine groups of carbons can be assigned at *τ*138.7, *α*136.4, *η*132.6, *θ*131.3, *χ*130.3, *ρ*128.0, *β*123.1, *σ*122.7, *φ*122.4–122.2. It should be noted that, the chemical shifts of protons and carbons change slightly with the oxidant/monomer ratio. Particularly, the ^1^H and ^13^C NMR spectra of the PFA obtained at the oxidant/monomer molar ratio of 3 are relatively simple, but those of the other three PFAs synthesized at the oxidant/monomer molar ratios of 5 to 9 are very complex. It seems that the ^1^H and ^13^C NMR spectra of the PFAs obtained at the oxidant/monomer ratios of 5 to 9 are the addition of the spectra of the PFAs synthesized under optimal conditions and at the oxidant/monomer ratio of 3, indicating that the FA polymerization significantly depends on the polymerization conditions. It is interesting to note that the *χ* protons tend to appear at lower fields from 8.52 to 8.56 to 8.58 ppm when changing the oxidant/monomer molar ratio from 3 to 9 to optimal. On the basis of the macromolecular structure of PFA, it appears that the chemical shifts of the *χ* protons could be used to evaluate the large π-conjugation of the PFA. That is to say, the polymer with the *χ* protons located at higher chemical shifts corresponds to higher molecular weight. This indicates that the optimal PFA indeed has the highest molecular weight, which is consistent with the UV-vis spectra demonstrating the strongest band III in Fig. 1f and elemental analyses having a higher C/H ratio in Table 2.

**Fig. 6:**
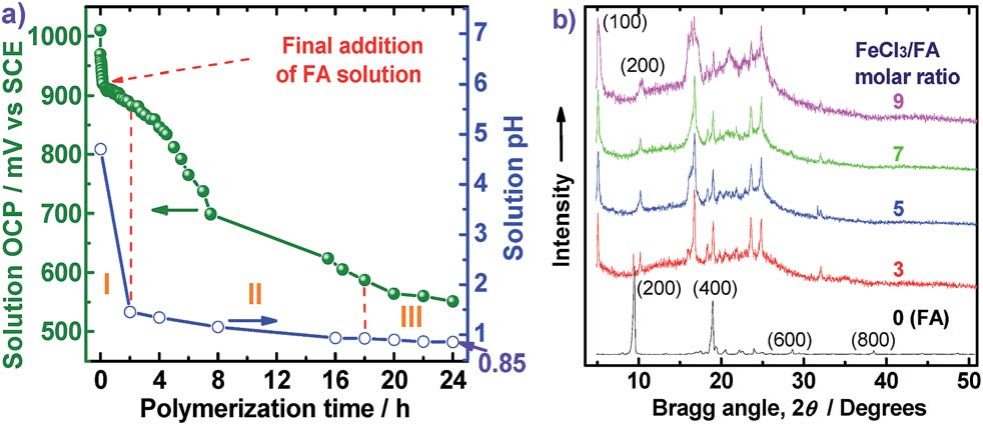
(a) The open-circuit potential (OCP) and pH variations of the FA polymerization solution with the polymerization time at a fixed FeCl_3_ (28 mmol)/FA (4 mmol) molar ratio of 7 in C_6_H_14_/CH_3_NO_2_ with a volume ratio of 30 mL/20 mL at 50 °C. The FA solution in C_6_H_14_ has initial OCP and pH of 165 mV *vs.* SCE and 5.6, respectively. The orange Roman numerals indicate the three polymerization stages. (b) Wide-angle X-ray diffractograms of the FA monomer and PFA synthesized in C_6_H_14_/CH_3_NO_2_ with a volume ratio of 30 mL/20 mL at 50 °C with four FeCl_3_/FA molar ratios.

The OCP and pH variations of the FA polymerization solution with reaction time are shown in Fig. 6a, providing insight into three polymerization stages: (I) fast polymerization, (II) moderate polymerization, (III) slow polymerization. The OCPs of FA monomer and FeCl_3_ oxidant solutions are 165 and 1010 mV *vs.* SCE, respectively. In the first stage, with the drop-wise addition of FA solution, the OCP of FeCl_3_ solution decreases rapidly from 1010 to 912 mV *vs.* SCE within the first 20 min because of a fast consumption of the oxidant to form active FA monomer carbonium ions and polymers. The pH of the reaction system also sharply declines from 5.6 to 4.5 due to the production of HCl during the oxidative dehydrogenation of FA monomers. In the second stage between 2 and 18 h after all the FA solution was added, the OCP and pH of the biphasic system further decreased from 883 to 587 mV *vs.* SCE and 1.45 to 0.92, respectively. Such significant decreases of OCP and pH can be attributed to moderate polymerization between active FA monomers and oligomers to form higher molecular weight polymers, as shown in Fig. 2b. In the third stage between 18 and 24 h, both OCP and pH decreased slowly with polymerization time, suggesting that a few residual monomers or oligomers or even the polymers are further oxidized increasing the molecular weight of the polymers. In other words, the cationic oxidative polymerization of FA has essentially finished and a very small amount of PFA polymer may be over-oxidized. As a result, both the polymerization yield and π-conjugation degree becomes lower at 24 h, as depicted in Fig. 2b. It appears that elevating the polymerization temperature from 50 °C to 70 °C could change reactive sites from *χ* into *α* positions. This investigation into the OCP and pH of FA polymerization solution strongly suggests that a polymerization time of 20 h is optimal.

**Scheme 2:**
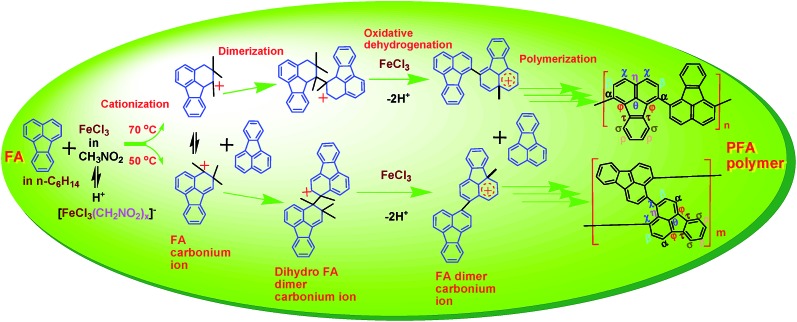
Cationic oxidative dehydrogenation polymerization of FA in hexane by FeCl_3_ in CH_3_NO_2_.

The FA polymerization does not appear to follow a radical mechanism because the FA monomer in CH_3_NO_2_ does not polymerize in the presence of (NH_4_)_2_S_2_O_8_, a typical initiator for radical polymerization. The FA polymerization has an oxidative feature because the pH of the reaction solution steadily decreased with reaction time as shown in Fig. 6a, which originates from dehydrogenating oxidation of active FA propagating chains by Fe^3+^. At the same time, Fe^2+^ that resulted from the reduction of Fe^3+^ was detected. Therefore, we believe that along with the FeCl_3_ catalyst, CH_3_NO_2_ can act as a cocatalyst by forming a H^+^[FeCl_3_(CH_2_NO_2_)_*x*_]^–^ complex that can attack FA monomer to form reactive FA carbonium ions, leading to initiation of the cationic oxidative polymerization of FA. A possible mechanism and reaction intermediates for the cationic oxidative polymerization of FA are proposed in Scheme 2, analogous to the polymerization of pyrene, naphthalene, and triphenylene by FeCl_3_.^23,30,31^ In particular, the polymerization consists of three elementary reactions:

(1) Chain initiation involving two steps: (a) fast and easy formation of the H^+^[FeCl_3_(CH_2_NO_2_)_*x*_]^–^ resulting from complexation of FeCl_3_ with H^+^(CH_2_NO_2_)^–^ originating from a tautomerism between CH_3_NO_2_ and H^+^(CH_2_NO_2_)^–^ which in turn acts as a pseudo-acid with a p*K*_a_ of 10.2, and (b) the difficult formation of FA carbonium ions because no sufficiently reactive FA monomers could be attacked by the H^+^[FeCl_3_(CH_2_NO_2_)_*x*_]^–^ until the temperature was elevated up to 70 °C or the FeCl_3_/FA ratio was increased to 7. Under either of these conditions dark PFA polymer precipitates formed immediately upon mixing FA monomer in hexane with FeCl_3_ in nitromethane, and thus, the PFA polymer with both high polymerization yield and high π-conjugation degree can be accomplished, as revealed in Fig. 2a and c. It appears that the cationic oxidative polymerization depends largely on the formation of the active FA carbonium ions. Only higher temperatures (>70 °C) can promote the formation of an adequate amount of active FA carbonium ions to achieve fast chain propagation. In other words, chain initiation is the rate-determining step.

(2) Fast chain propagation including dimerization, trimerization, tetramerization, *etc.* by inserting FA monomer between the corresponding carbonium ions and counter anion [FeCl_3_(CH_2_NO_2_)_*x*_]^–^, followed by oxidative dehydrogenation condensation in the presence of FeCl_3_ oxidant at a sufficiently high concentration, leads to (a) dark PFA polymer precipitates formed immediately upon mixing FA monomer with FeCl_3_ in nitromethane and (b) no significant variation of the π-conjugation degree of the PFA formed with increasing the reaction time from 2 to 24 h, as demonstrated in Fig. 2b. Moreover, the H^+^ released during the oxidative condensation chain propagation of the active oligomer carbonium ions will promote the formation of H^+^[FeCl_3_(CH_2_NO_2_)_*x*_]^–^, which is beneficial to the formation of more active FA carbonium ions. That is, the FA polymerization possesses self-catalyzing characteristics.

(3) Termination of active PFA carbonium chains by deprotonation.

It can be concluded that cationic oxidative dehydrogenation polycondensations are likely in forming the resulting PFAs.

On the basis of the steady pH reduction of the polymerization solution shown in Fig. 6a, the degree of dehydrogenation of the FA monomer can be calculated. In this study, the apparent polymerization yield was 85 wt%, implying that at least 85% × 4 mmol ≥3.4 mmol of FA monomers participate in dehydrogenation. After the polymerization for 24 h, the pH of the final solution was found to be 0.85, as indicated in Fig. 6a. As a result, the H^+^ concentration in the reaction system was 10^–0.85^ = 0.14 M, *i.e.*, 0.14 M × 50 mL = 7 mmol hydrogen protons were released because of dehydrogenation. Therefore, the average dehydrogenation number in one FA monomer is 7 mmol/3.4 mmol ≤ 2.06, which is close to 2.0 for a helix macromolecular structure of PFA as summarized in Table 2.

WAXD powder patterns of FA monomer and PFAs synthesized with various FeCl_3_/FA ratios are shown in Fig. 6b. The FA monomer exhibits two very strong diffraction peaks at 9.48° (*d*_200_ 0.932 nm, strongest) and 18.96° (*d*_400_ 0.468 nm), as well as eight very weak peaks at 19.30° (*d*_–204_ 0.460 nm), 23.96° (*d*_–214_ 0.371 nm), 20.50° (*d*_004_ 0.433 nm), 22.20° (*d*_–313_ 0.400 nm), 22.54° (*d*_113_ 0.394 nm), 28.62° (*d*_600_ 0.308 nm), and 38.48° (*d*_800_ 0.234 nm), while the PFAs show two groups of diffraction peaks at 5.06° (*d*_100_ 1.744 nm)/10.16° (*d*_200_ 0.870 nm), and 19.78° (0.448 nm)/24.86° (0.358 nm)/9.38° (0.942 nm)/16.78° (*d*_012_ 0.528 nm strongest)/19.02° (0.466 nm)/23.6° (0.377 nm), suggesting that there are two kinds of crystalline ordered structures in the PFAs, although they also present a broad diffraction in the region of 15–30° due to an amorphous non-crystalline state of irregular lateral suprastructure of the PFA molecules regardless of the FeCl_3_/FA ratio used for their synthesis. A series of *d*_100_/*d*_200_ diffractions indicate that the PFAs have crystalline order that is different from the crystalline structure as FA monomer displaying a series of *d*_200_/*d*_400_ diffractions because their strongest diffraction peaks are rather different. This crystalline order may be a layered crystalline structure with an interlayer *d* spacing of 1.744 nm that would be a typical intermolecular π–π stacking distance. It seems that the crystalline diffraction peaks gradually become stronger when the oxidant/monomer molar ratio is decreased from 9 to 0 and finally the FA and PFA obtained especially at the oxidant/monomer molar ratio of 3 have the same diffraction peaks at 9.38° and 19.02°, suggesting that the PFAs consist of FA repeat units and also the FA unit structure was not broken during the oxidative polymerization. It is well known that most chemical oxidative polymers including polypyrrole,^32^ polyaniline,^33^ polythiophene,^17^ and polyfuran^34^ result in amorphous materials. However, the PFAs synthesized at the biphasic interface between C_6_H_14_ and CH_3_NO_2_ have a combination of crystalline and non-crystalline structures since the molecular weight of the PFAs synthesized in this study is not high enough. It should be noted that all the PFA powder prepared by various FeCl_3_/FA ratios illustrates a very weak diffraction around 32° that may be assigned to the characteristic peak of FeCl_3_,^17^ although the samples were thoroughly purified before the X-ray diffraction measurements. According to previous reports,^35,36^ the PFA would be able to form a salt (FA unit)_2_X with an anion of FeCl_4_^–^, like the FA monomer.

PFA particles can be uniformly re-dispersed in DI water, forming a red suspension by sonication, but tending to aggregate after 30 min of standing. The size distribution of the pure optimal PFA particles in DI water was analyzed by LPA in Fig. S1a.† The result shows that the number-average diameter and size polydispersity of PFA particles are 6.79 μm and 1.18, respectively, indicating that the PFA particles are basically uniform regardless of their aggregation tendency in water because of their highly hydrophobic character. Fortunately, the PFA particles can simply be dispersed in nonaqueous organic solvents such as ethanol, acetonitrile, acetone, and CHCl_3_, forming a very stable dispersion. For example, PFA particles form a uniform red dispersion in ethanol and do not precipitate even after one day of standing. The size distribution of PFA particles prepared with various C_6_H_14_/CH_3_NO_2_ ratios analyzed by DLS is depicted in Fig. S1b.† By changing the C_6_H_14_/CH_3_NO_2_ volume ratio from 1/1, 3/2, 3/1, to 50/3, the size of the obtained PFA after 30 min sonication varies from 524 to 217 to 216 to 243 nm, respectively. This has been confirmed by the SEM image in Fig. S1c† that signifies a similar size of the PFA particles of around 220 nm if synthesizing in the C_6_H_14_/CH_3_NO_2_ volume ratio of 3/2. In particular, the PFA synthesized in the C_6_H_14_/CH_3_NO_2_ volume ratio of 3/2 has the diameter and the narrowest size distribution, whereas the PFA synthesized in the C_6_H_14_/CH_3_NO_2_ ratio of 3/1 has the smallest size. Clearly, the C_6_H_14_/CH_3_NO_2_ volume ratios of 3/2 and 3/1 are the best to obtain PFA submicron particles. Note that the remarkably different sizes can be mainly attributed to the different dispersion states of PFA particles in water and acetone. That is to say, the dispersion of PFA particles in acetone is much better than in water.

### Properties of PFA

#### Solubility and solvatochromism

As listed in Table S3,† although FA monomer is soluble in nearly all organic solvents including HCOOH, NMP, DMSO, DMF, CH_3_CN, CH_3_NO_2_, CHCl_3_, THF, CH_3_COOH, and benzene, the PFAs synthesized in this study are only soluble in NMP and DMSO with strong polarity, partly soluble in DMF and 98% H_2_SO_4_, and almost insoluble in other solvents. At the same time, the PFAs can disperse very well in HCOOH, CH_3_CN, CH_3_NO_2_, CHCl_3_, and THF, as mentioned above. The solubility of PFAs also depends on their synthetic conditions. For instance, the PFA synthesized with FeCl_3_/FA molar ratios of 3, 5, and 9 presents the highest solubility, but PFA synthesized with an FeCl_3_/FA molar ratio of 7 has a lower solubility possibly due to a higher degree of π-conjugation (Fig. 2c). Furthermore, the optimal PFA exhibits the lowest solubility because of its highest degree of π-conjugation. The tunable solubility and dispersibility of PFA makes itself more useful because solubility is good for processing, while insolubility means strong chemoresistance. Meanwhile, the PFAs display colorful solvatochromism. As an example, the solution of the optimal PFA exhibits red, red, brilliant orange, dark green, and pink in NMP, DMSO, DMF, H_2_SO_4_, and CHCl_3_, respectively. Additionally, the PFAs synthesized with different FeCl_3_/FA molar ratios also exhibit colorful solvatochromism (Table S3†). The solvatochromism might originate from different conformations and then different π–π conjugation lengths in different solvents. The doping effect of proton acids like H_2_SO_4_ and HCOOH may be responsible for the green solvatochromism that is comparable to green acid-doped polyaniline.^37^

#### Strong and visible fluorescence

**Fig. 7:**
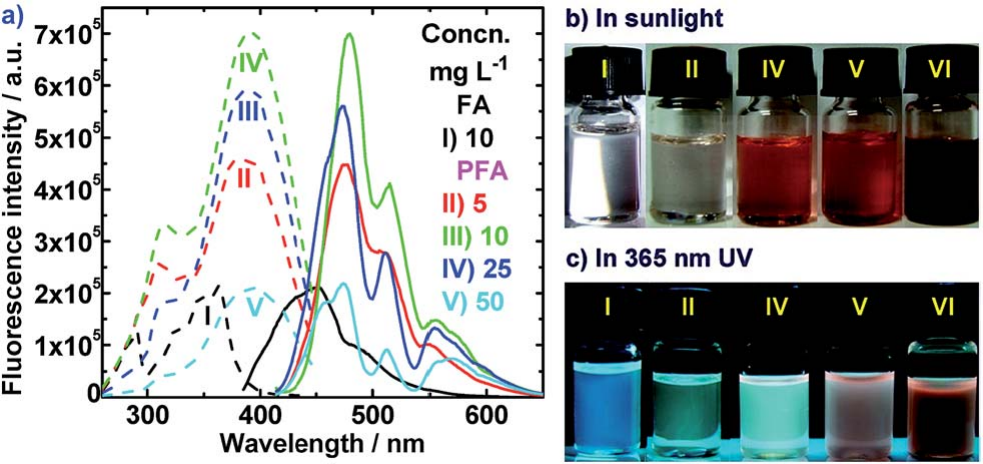
(a) Fluorescence excitation spectra (dashed lines) taken at (I) 452 nm, (II) 472 nm, (III) 478 nm, and (IV–V) 474 nm emitting and fluorescence emission spectra (solid lines) taken at (I) 363 nm, and (II–V) 400 nm exciting. (b) DMSO solution in sunlight and (c) under 365 nm UV for (I) 10 mg L^–1^ FA and the optimal PFA at different concentrations: (II) 5, (III) 10, (IV) 25, (V) 50, and (VI) 50 mg L^–1^.

Fluorescent excitation and emission spectra of FA and optimal dedoped PFA solutions in DMSO are shown in Fig. 7a. It is observed that the PFA exhibits 3.3-times stronger excitation and emission than FA monomer, whereas the corresponding enhancement is only 2 times upon electropolymerization of FA monomer.^16^ The maximum excitation of PFA corresponds to a much longer wavelength (392 nm) than that of FA monomer (363 nm). This implies that the PFA needs much lower energy to excite the fluorescence than FA monomer. Similarly, PFA also exhibits a much longer emission wavelength (peaked at 480 nm) than FA monomer (peaked at 452 nm), indicating that the emission from the PFA is more visual. On the other hand, it can be clearly seen that the emission spectra of all the PFAs show triple bands at 480, 515 and 556 nm due to π* electronic transitions which arise from an average distribution of large π-conjugation lengths of conjugated polymers. The maximum emission wavelength of 10% PFA solution shows an 88 nm red shift as compared with the maximum excitation wavelength, which is close to the 90 nm red shift observed for electropolymerized PFA^16^ and the 89 nm red shift of FA. The fluorescent intensity of the PFA significantly increases at first and then decreases with augmenting the PFA concentration from 5 to 50 mg L^–1^, giving the maximum intensity at 10 mg L^–1^. The excitation and emission spectra of PFA have smaller half-widths and higher maximum excitations and emission wavelengths than the FA monomer. All of this information confirms that PFA has a large π-conjugated structure because the fluorescence properties are greatly improved upon chemical oxidative polymerization of FA.

As expected, in agreement with the fluorescence spectra, distinct fluorescence differences between FA and PFA in DMSO under a 365 nm UV light are observed. In sunlight, the FA and PFA solutions in Fig. 7b and S2† are colorless and red, respectively. Upon excitation with a 365 nm UV lamp, the FA solution emits little differentiable fluorescence light in Fig. 7c and S2,† but the PFA solution emits a strong, visible skyblue fluorescence.^38^ Furthermore, the fluorescence of PFA largely depends on the FeCl_3_/FA ratio in Fig. S2 (middle),† PFA concentration in Fig. 7c and S2 (bottom),† solvents and UV wavelength used. At the same concentration, the solution of PFA synthesized with a FeCl_3_/FA molar ratio of 7 emits the brightest fluorescence. This implies that the PFA obtained at the FeCl_3_/FA molar ratio of 7 has the largest degree of conjugation, as confirmed earlier. With increasing PFA concentration from 5 to 500 mg L^–1^, the solution of the optimal PFA first emits an increased and then a decreased fluorescence, realizing the strongest fluorescence at 25 mg L^–1^, which can be seen in Fig. 7c. Note that the fluorescence emission would become weaker if using concentrated H_2_SO_4_ or excitation at 254 nm. This excellent fluorescence and its sensitivity to PFA concentration are very important for its potential applications in OLEDs and other optoelectronic devices.

**Fig. 8:**
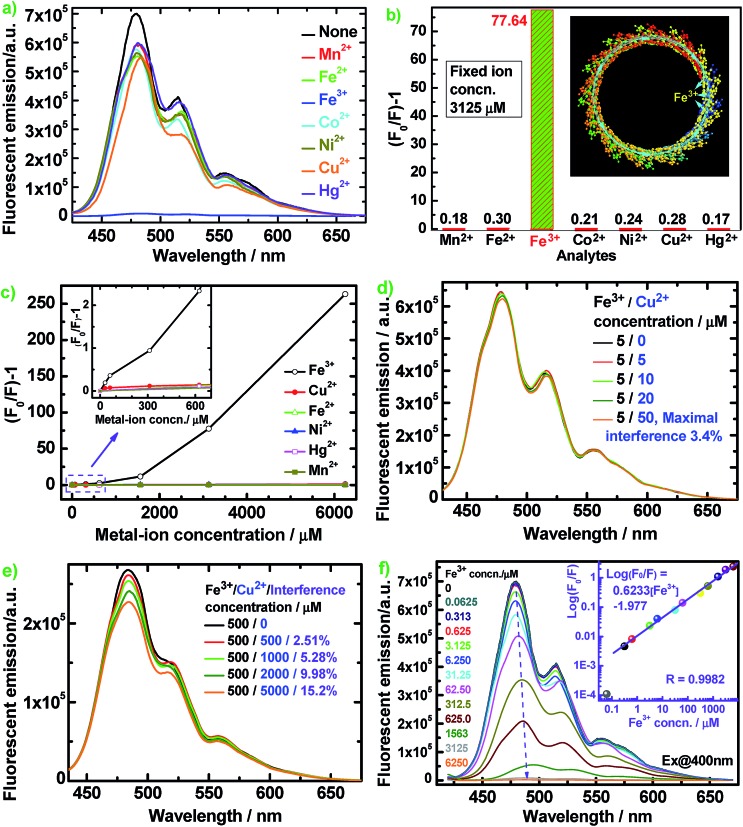
(a) Fluorescence emission spectra and (b) (*F*_0_/*F*) – 1 values of 10 mg L^–1^ optimal PFA in DMSO containing different metal ions at a fixed concentration of 3125 μM (inset: schematic diagram of the amplified quenching of PFA fluorescence by Fe^3+^); (c) relationship between (*F*_0_/*F*) – 1 and metal-ion concentration of 10 mg L^–1^ optimal PFA in DMSO; (d and e) the influence of coexisted Cu^2+^ on the PL emission of the optimal PFA solution containing Fe^3+^; (f) PL emission spectra of 10 mg L^–1^ optimal PFA in DMSO containing Fe^3+^ of 0 to 6250 μM.

Fig. 8 illustrates the diverse changes of the fluorescence emission of the optimal PFA upon the addition of seven metal ions in aqueous solution. The fluorescence is slightly quenched by Mn^2+^, Fe^2+^, Co^2+^, Ni^2+^, Cu^2+^, and Hg^2+^, but is almost completely quenched only by Fe^3+^ at the same ion concentration of 3125 μM in Fig. 8a–c. As a typical example, Fig. 8d reveals that the interference of coexisting Cu^2+^ (≤50 μM) on the fluorescent emission of optimal PFA solution containing 5 μM Fe^3+^ is less than 3.42%. Similarly, the interference of coexisting Cu^2+^ (≤1000 μM) on the emission of optimal PFA solution containing 500 μM Fe^3+^ is less than 5.28%, as shown in Fig. 8e. It should be noted that coexisting Cu^2+^ at ≥2000 μM would interfere with the emission of optimal PFA solution containing 500 μM Fe^3+^ to some extent. All these examples indicate that the selectivity of the PFA toward Fe^3+^ over the other six ions is extraordinarily high as long as the other six ions do not have a concentration four times higher than Fe^3+^. This suggests that PFA definitely has high selectivity toward Fe^3+^ over the other six ions. Note that (*F*_0_/*F*) – 1 increases significantly and nonlinearly with Fe^3+^ concentration, indicating that energy transfer along the PFA π-conjugation enlarges the quenching degree of Fe^3+^.^39^

Fig. 8f shows that the fluorescent emission maximum of PFA containing 1563 μM Fe^3+^ is red-shifted by about 15 nm, indicating a strong interaction between Fe^3+^ and PFA chains. Log(*F*_0_/*F*) *vs.* Fe^3+^ concentration illustrates a good linear relationship with a correlation coefficient (*R*) of 0.9982 over a wide Fe^3+^ concentration range of 0.313 to 6250 μM with a detection limit down to 62.5 nM, producing the equation log(*F*_0_/*F*) = 0.6233[Fe^3+^] – 1.977 (Fig. 8f, inset). Without the requirement for an organic solvent, PFA solution can be used for the direct selective detection of Fe^3+^ in an aqueous sample. Thus PFA is a new, important and sensitive fluorescent substance for highly and selectively responsive Fe^3+^ detection. Fe^3+^ has many more outer empty orbitals and sub-outer semi-empty orbitals than the other six ions. As a result, the electron deficient Fe^3+^ strongly interacts with the greatly electro-donating large π-conjugated PFA, where a schematic of the π-electron transfer complex with the quencher Fe^3+^ is presented where the cyan dotted lines represent the π-electrons and cyan arrows represent the flow directions of the π-electrons, as shown in Fig. 8b inset. The overwhelmingly amplified quenching effect is most likely related to energy or electron transfer from the PFA to the Fe^3+^ complex, which is a non-radiative center and traps the excitation energy.

#### High thermal stability

As discussed above, PFA shows well-developed layer crystalline structures that can melt at a relatively high temperature. It can be seen that FA begins to melt at 110 °C, but the PFAs do not melt until 295 °C because the PFAs have much higher molecular weights. The melting temperature of the PFAs increases first and then decreases with increasing oxidant/monomer molar ratio from 3 to 9, reaching a maximum at the oxidant/monomer molar ratio of 7. Moreover, the optimal PFA has the highest melting temperature of up to 380 °C owing to its highest molecular weight.

**Fig. 9:**
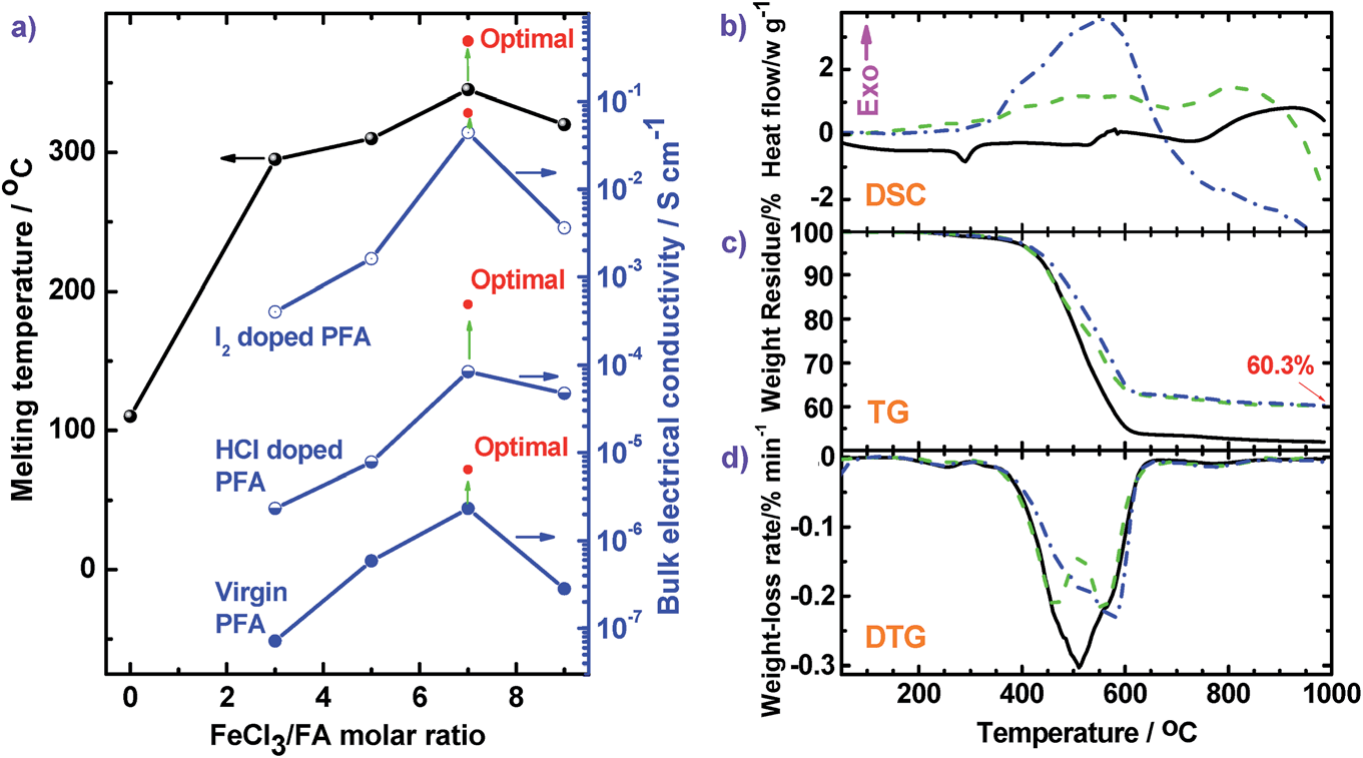
(a) Melting temperature and conductivities of FA and PFA synthesized with various FeCl_3_/FA molar ratios and under optimal conditions. (b) DSC, (c) TG, and (d) DTG curves of PFAs synthesized at different polymerization temperatures: (black solid) 50 °C and FeCl_3_/FA molar ratio of 5 for 24 h; (green dash) 70 °C and FeCl_3_/FA molar ratio of 5 for 24 h; (blue dash dot) 70 °C and FeCl_3_/FA molar ratio of 7 for 18 h. Other synthetic conditions were fixed: drop-wise addition of FA to FeCl_3_ solutions and C_6_H_14_/CH_3_NO_2_ volume ratio of 3/2.

The thermal properties of three types of PFAs were further analyzed by DSC, TG, and DTG (Fig. 9). The thermograms do not show any obvious thermal transition below 250 °C, signifying that small molecule impurities such as H_2_O and HCl were thoroughly removed. DSC scans (Fig. 9b) of the PFA (a) show an endothermic peak around 290 °C due to melting behavior. Three DSC scans all exhibit exothermic peaks around 551–590 °C because of thermal decomposition of the rings of the PFAs. This exothermic decomposition is confirmed by TG scans which display major weight loss at 400 to 600 °C. The TG scans reveal that the PFA-based char is very thermally stable because the weight-loss is less than 2% when the temperature is further elevated from 625 to 985 °C. The char yield at 985 °C increases from 51.9 to 60.3% when the polymerization temperature is elevated from 50 °C (solid line) to 70 °C (dash line) because the PFA synthesized at 70 °C has a larger degree of conjugation and higher molecular weight. According to the DTG curves, by increasing the polymerization temperature from 50 °C (solid line) to 70 °C (dash line), the temperature (*T*_dm_) at the maximal weight-loss rate ((d*α*/d*t*)_m_) rises from 510 to 557 °C, with corresponding (d*α*/d*t*)_m_ decreases from 0.303 to 0.219% min^–1^. Meanwhile, the PFA obtained with a FeCl_3_/FA molar ratio of 7 demonstrates the highest *T*_dm_ of 576 °C and the second lowest (d*α*/d*t*)_m_ of 0.231% min^–1^. This information confirms the great advantages of using chemical oxidative polymerization to facilely synthesize high quality PFA with higher purity, higher carbon yield, and higher thermo- and chemo-resistances than electropolymerization. For example, the electropolymerized PFA exhibits a high impurity content up to 14.6%, low *T*_dm_ down to 442 °C, and char yield down to 23.5% at 727 °C, respectively.^16^ In addition, the high molecular weight PFA obtained by the cost-effective chemical oxidative polymerization of FA is even more thermally stable than most typical heat-resistant polymers,^40–42^ except for expensive polybenzazole^43^ and poly(*p*-phenylene benzobisthiazole) (PBT)^44^ (Table S4†). In other words, PFA consisting of only polycyclic fused ring hydrocarbons is promising to be developed as a new highly thermally resistant material that is relatively inexpensive.

#### Tunable electrical conductivity

The bulk electrical conductivity of the PFA particles is strongly influenced by the polymerization conditions and their doping states although FA monomer is an insulator in any state. The conductivity of pristine PFA significantly varies with the FeCl_3_/FA molar ratio from 0 to 9, as summarized in Fig. 9a, exhibiting a maximum conductivity of 2.3 × 10^–6^ S cm^–1^ at the FeCl_3_/FA molar ratio of 7, which exactly corresponds to the maximum π-conjugation degree in Fig. 2c. The decreased conductivity at the higher FeCl_3_/FA molar ratio of 9 could result from over-oxidation. The red inset point in Fig. 9a suggests that optimal pristine PFA has the highest conductivity of 6.4 × 10^–6^ S cm^–1^ because of its highest molecular weight and largest degree of conjugation. Significantly, the conductivity of all the PFAs increase by 2–4 orders of magnitude upon doping with concentrated HCl or I_2_ vapor. I_2_ doped optimal PFA possesses much higher conductivity of up to 0.072 S cm^–1^, which is 150 times higher than that of HCl doped PFA. The largely enhanced conductivity of PFA after I_2_ doping is in accordance with pentacene^45^ and poly(perinaphthalene)^46^ which share similar structures with PFA. Note that the conductivity can be further enhanced by thermal treatment of pristine PFA at high temperature. Three carbonized samples in nitrogen at up to 985 °C have much higher conductivities of 5.2, 8.5, and 11.6 S cm^–1^ for the PFAs (a), (b), and (c), respectively (Fig. 9). Surprisingly, when the optimal PFA was carbonized in argon at 1100 °C for 30 min, the conductivity of the PFA-based carbon particles increased to as high as 150 S cm^–1^ with a carbon yield of 50%. The well developed structure of these highly conducting carbon particles has been confirmed by two typical Raman bands of carbon: the strongest band at 1306 cm^–1^ and the sharpest Raman band at 1584 cm^–1^ due to D- and G-bands, as shown in Fig. 3b. Therefore, the conductivity of PFA could be efficiently optimized by controlling and regulating the polymerization, doping, as well as heat treatment.

## Conclusions

We have synthesized multifunctional polyfluoranthenes with a helical configuration by a facile cationic oxidative polymerization of fluoranthene at the interface between C_6_H_14_ and CH_3_NO_2_ using FeCl_3_ as catalyst and oxidant. The effects of the polymerization parameters including polymerization medium, C_6_H_12_/CH_3_NO_2_ ratio, method of oxidant/monomer mixing, oxidant/monomer ratio, polymerization temperature and time on polymerization yield, macromolecular structure, particle size distribution, solubility, solvatochromism, electrical conductivity, fluorescence, and thermal properties have been systematically optimized. Optimal synthesis of PFA possessing the highest polymerization yield up to 94%, most extensive π-conjugation, highest molecular weight, and highest electrical conductivity can be obtained as follows: drop-wise addition of FA solution to FeCl_3_ solution, C_6_H_12_/CH_3_NO_2_ volume ratio of 3/2, polymerization temperature of 70 °C, polymerization time of 18 h, and FeCl_3_/FA molar ratio of 7. This polymer exhibits a wide range of controllable conductivity from 7.1 × 10^–8^ S cm^–1^ to 150 S cm^–1^, good solubility, unique solvatochromism, high thermostability and relatively high char yield at 985 °C. In particular, this polymer exhibits excellent visible skyblue fluorescence emission and high selective response for detecting Fe^3+^ with a detection limit down to 62.5 nM because of the synergistic effect of well-distributed π-conjugated electrons throughout the polyfluoranthene which function as both the fluorophore and the receptor units simultaneously, avoiding complicated multistep procedures of other superamplified fluorescent polymers that usually contain bulky fluorophore groups and specific receptors. This new PFA is a promising material for use as a strongly fluorescent emitting organic material and a selectively sensitive Fe^3+^ chemosensor, an organic semiconductor, and a cost-effective carbon precursor.
