# Supplementary material for: Interfacial chemical oxidative synthesis of multifunctional polyfluoranthene
Source: Chem Sci. 2015 Jan 21;6(3):2087–101. doi: 10.1039/c4sc03890h (PMC5654415; doi:10.1039/c4sc03890h)
Supplement: Supplementary file 1 [file SC-006-C4SC03890H-s001.pdf]

Electronic Supplementary Material (ESI) for Chemical Science.
This journal is © The Royal Society of Chemistry 2015

Interfacial chemical oxidative synthesis of multifunctional polyfluoranthene

Xin-Gui Li,‡*ab Yao-Zu Liao,‡ab Mei-Rong Huang*ab and Richard B. Kaner*b

Electronic supplementary information

Table S1. Characteristics of Virgin PFA Synthesized by Three Different Feed Methods[a]

Reactant Feed Method	PFA product color	Polymerization yield / %	UV-vis wavelength in NMP / nm			Large π-conjugation degree: intensity ratio of band III to band I
			Band I	Band II	Band III	
Drop-wise addition of $FeCl_3$ into FA	Dark yellow	60.5	292	502	537	0.083
Direct mixing	Dark red	68.9	292	500	535	0.297
Drop-wise addition of FA into $FeCl_3$	Brown	72.4	291	500	533	0.352

[a] The same fixed conditions of C_6H_{14}/CH_3NO_2 volume ratio of 3/2, $FeCl_3$/FA molar ratio of 5, polymerization temperature of 50 °C, and polymerization time of 24 h.

Table S2. Characteristics of Virgin PFA Synthesized with Various C_6H_{14}/CH_3NO_2 Volume Ratios[a]

C_6H_{14}/CH_3NO_2 volume ratio	PFA product color	Polymerization yield / %	UV-vis wavelength in NMP/nm			Large π-conjugation degree: intensity ratio of band III to band I
			Band I	Band II	Band III	
2/3	Brown	86.5	262	501	536	0.432
1/1	Black	87.6	265	500	535	0.465
3/2	Black	88.1	262	500	531	0.509
4/1	Brown	81.3	292	500	535	0.401

[a]Fixed conditions: Oxidant $FeCl_3$/monomer FA molar ratio of 7 at 70 °C for 24 h.

aState Key Laboratory of Pollution Control and Resource Reuse, College of Environmental Science and Engineering, Tongji University, 1239 Si-Ping Road, Shanghai 200092, China.
E-mail: adamxgli@yahoo.com; huangmeirong@tongji.edu.cn; Fax: +86-21-65983869; Tel: +86-21-69582104
bDepartment of Chemistry & Biochemistry and California NanoSystems Institute, University of California, Los Angeles, Los Angeles, California 90095, USA. E-mail: kaner@chem.ucla.edu; Fax: +1 (310) 206-4038, Tel: +1 (310) 825-5346
‡ X.-G. Li and Y. -Z. Liao contributed equally to this work.

Table S3. Solubility and Solution Color of PFA Synthesized with Various $FeCl_3$/FA Molar Ratios[a]

$FeCl_3$/FA molar ratio	Solubility[b] and solution color[c] of FA and PFA in various solvents										
	NMP	DMSO	DMF	98%H_2SO_4	HCOOH	CH_3CN	Benzene	CH_3COOH	CH_3NO_2	$CHCl_3$	THF
0 (FA)	S, cl	S, cl	S, cl	PS, g	S, cl	S, cl	S, cl	S, cl	S, by	S, cl	S, cl
3	S, bo	S, bo	PS, y	PS, dg	SS, gg	IS	MS, y	PS, p	IS	IS	IS
5	S, r	S, bo	PS, bo	PS, dg	SS, gg	IS	MS, y	PS, p	IS	IS	IS
7	S, r	MS, r	PS, bo	PS, dg	SS, gg	IS	MS,bo	PS, p	IS	IS	IS
9	S, r	S, lr	PS, bo	PS, dg	SS, gg	IS	MS, o	PS, p	IS	IS	IS
Optimal	MS, r	MS, r	PS, bo	PS, dg	IS	IS	MS, lr	PS, p	IS	SS, p	IS

[a]Fixed conditions: C_6H_{14}/CH_3NO_2 volume ratio 3/2, polymerization temperature 50 °C, and polymerization time 18 h.

[b]IS=Insoluble; MS=mostly soluble; PS=partially soluble; S=soluble; SS=slightly soluble.

[c]bo=brilliant orange; by=brilliant yellow; cl=colorless; dg=dark green; g=green; gg=grassy green; lr=light red; o=orange; p=pink; r=red; y=yellow.

Table S4. Thermal Properties of PFAs and Selected Heat Resistant Polymers

Polymers	Atmosphere	Heating rate (°C·min^{-1})	T_d / T_{dm} (°C)	$(d\alpha/dt)_m$ (wt%·min^{-1})	Char yield (% °C^{-1})	Refs.
**PFA[a]**	**N_2**	**20**	**420/510**	**0.303**	**52/985**	**This study**
**PFA[b]**	**N_2**	**20**	**422/557**	**0.219**	**60/985**	
**Optimal PFA**	**N_2**	**20**	**434/576**	**0.231**	**60/985**	
Poly(anthracene oil)	N_2	10	200/250	0.07	37/600	[40]
Poly(*o*-phenylenediamine)	N_2	10	-/677	2.7	39/700	[41]
Poly(oxybenzoate-co-oxynaphthoate)	He	20	529/543	31	40/800	[42]
Polybenzazole	N_2	10	700/720	—	68/800	[43]
Poly(*p*-phenylene benzobisthiazole)	He	20	675/767	—	84/800	[44]

[a],[b]Synthetic conditions: Polymerization temperature of [a] 50 °C and [b] 70 °C at the fixed other conditions: C_6H_{14}/CH_3NO_2 volume ratio of 3/2, $FeCl_3$/FA molar ratio of 5, and polymerization time of 18 h.

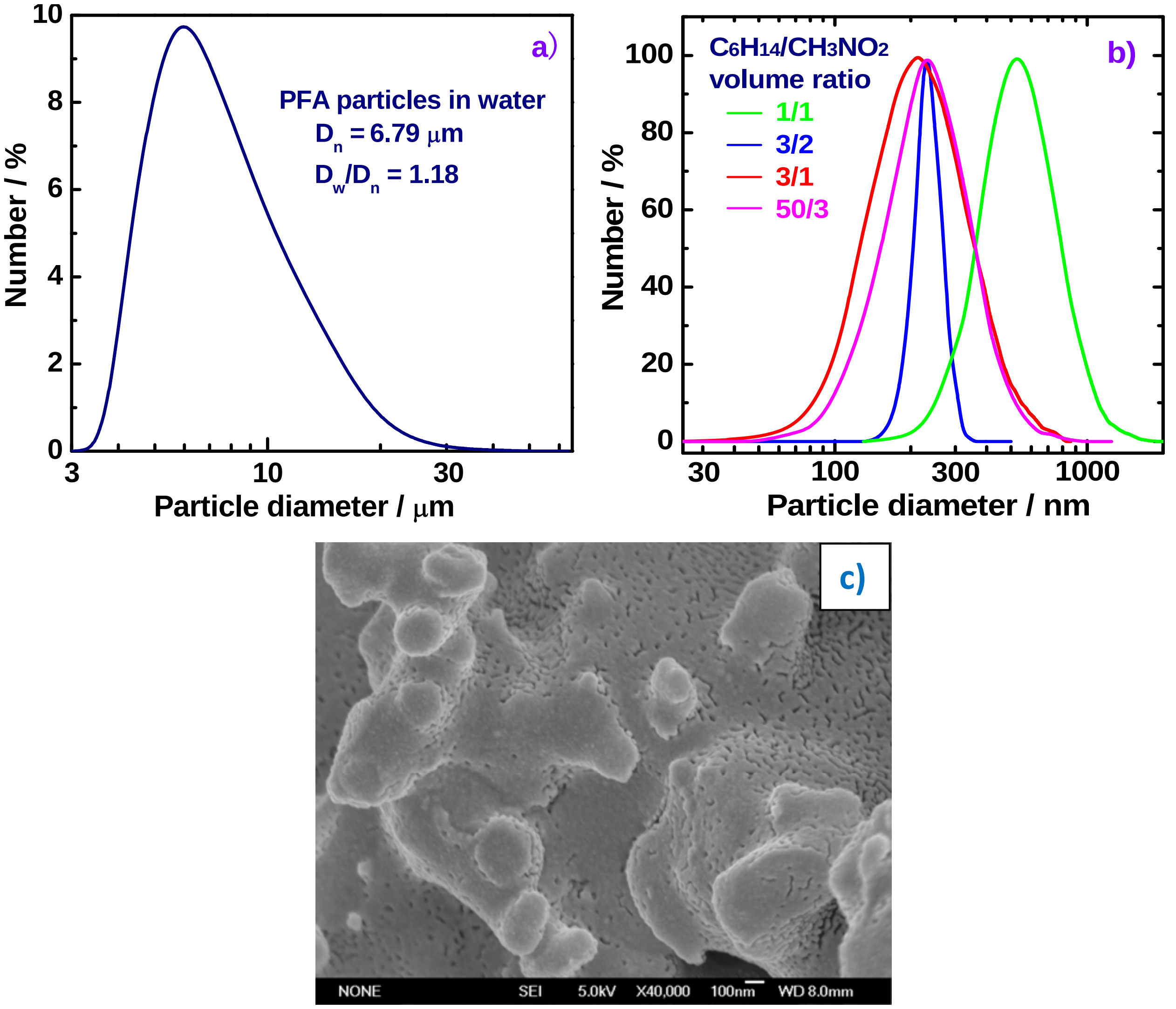

Fig. S1. Size distribution **a**) in pure water of the optimal PFA particles using a laser-particle size analyzer, **b**) in acetone determined by dynamic light scattering of the PFA particles synthesized with C_6H_{14}/CH_3NO_2 volume ratios of 1/1, 3/2, 3/1, and 50/3 at room temperature under the other optimal conditions of polymerization, and **c**) SEM image of the ethanol-dispersed PFA particles (with the diameter of around 220 nm) synthesized with a C_6H_{14}/CH_3NO_2 volume ratio of 3/2.

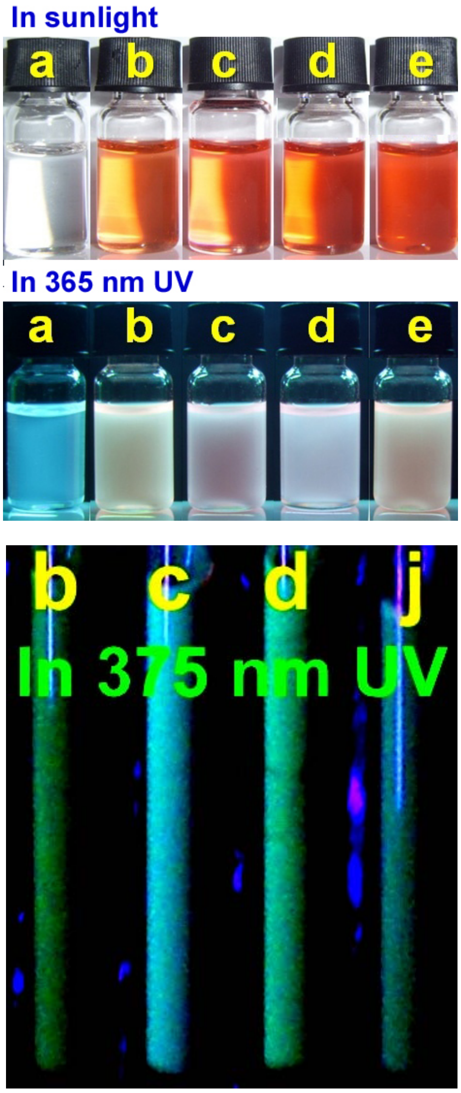

Figure S2. DMSO solution in sunlight (**top**) and in 365 nm UV (**middle**) of a) FA and PFA synthesized with various $FeCl_3$/FA molar ratios: b) 3, c) 5, d) 7, and e) 9 at a fixed concentration of 50 mg L^{-1}, optimal PFA at different concentrations: f) 5, g) 25, h) 50, i) 500 mg L^{-1}; (**bottom**) PFA synthesized with various $FeCl_3$/FA molar ratios of b) 3, c) 5, d) 7, and j) optimal PFA in 375 nm UV in DMSO-D6 at a very high concentration of ca. 10 g L^{-1}.
